# The Welfare Impact of Heat Stress in South American Beef Cattle and the Cost-Effectiveness of Shade Provision

**DOI:** 10.3390/ani16020231

**Published:** 2026-01-13

**Authors:** Cynthia Schuck-Paim, Wladimir Jimenez Alonso, Anielly de Paula Freitas, Camila Pereira de Oliveira, Vinicius de França Carvalho Fonseca, Tâmara Duarte Borges

**Affiliations:** 1 Center for Welfare Metrics, Sao Paulo 05508-090, Brazil; 2 Minerva Foods, Sao Paulo 04542-000, Brazil; 3 Instituto Federal Baiano, Santa Ines 45320-000, Brazil

**Keywords:** beef cattle welfare, Nelore, heat stress, shade, welfare footprint framework

## Abstract

Heat stress represents a pervasive welfare challenge for beef cattle and other species in tropical and subtropical regions. While its physiological and production impacts are well-documented, quantitative measures of the welfare impact of heat stress remain absent. This study provides the first quantification of the welfare impact of heat stress in beef cattle (mostly Nelore), estimated as cumulative time in thermal discomfort of four intensities (Annoying, Hurtful, Disabling, Excruciating) using the Welfare Footprint Framework. We analyzed climate data from 636 locations over five years across major beef production areas in Brazil, Argentina, Colombia, Paraguay, and Uruguay. Daily heat stress episodes and chronic heat stress exposure were assessed, respectively, using Comprehensive Climate Index (CCI) levels and the Annual Thermal Load metric, which sums daily excesses above a threshold of thermal comfort (CCI = 30 °C) throughout the year, classifying locations into five risk categories. Welfare impacts were estimated for thirteen heat stress scenarios modeled by considering each CCI level within each thermal risk category. Beef cattle in moderate-risk regions were estimated to experience primarily mild thermal discomfort for an average of 5 h daily. This duration increased to an average of 7 h daily in high-risk areas, of which 4.5 h in moderate to intense thermal discomfort (Hurtful or higher). Very high-risk regions reached 10 h of daily thermal discomfort, while extreme-risk regions showed beef cattle facing heat stress for over 11 h on 307 days annually, including over 3 h per day under severe thermoregulatory effort. Overall, 65% of animals were in regions of high thermal risk or above, experiencing between 280 and 2800 h annually in moderate to intense thermal discomfort—a magnitude that places heat stress among the most significant welfare challenges in animal production. Shade provision reduced time in severe discomfort of Disabling intensity by 85% (from 578 to 83 h annually), with economic returns of US$12–16 per animal and payback periods of approximately 16 months. By quantifying welfare impacts as cumulative time in thermal discomfort, shade provision emerges as one of the most effective welfare interventions available for beef cattle, and likely other grazing ruminants, in tropical and subtropical regions.

## Simple Summary

Heat stress is known to severely affect cattle welfare and productivity in tropical regions. This study provides the first quantification of the welfare impact of heat stress in beef cattle, measured as cumulative time spent in thermal discomfort of different intensities. Analyzing climate data from 636 locations across five South American countries (Brazil, Argentina, Colombia, Paraguay, Uruguay), we combined measures of daily environmental heat stress intensity (Comprehensive Climate Index, CCI) with chronic heat exposure (annual CCI excesses) to quantify welfare impacts. Across 65% of locations classified as high thermal risk or above, cattle were estimated to experience between 280 and 2800 h annually in moderate to intense thermal discomfort—a magnitude that places heat stress among the most significant welfare challenges in animal production. Shade structures were estimated to reduce time in intense thermal discomfort by 85% on average (from an average of 578 to 83 h annually), with payback within 16 months and net returns of US$12–16 per animal. By quantifying welfare impacts as cumulative time in thermal discomfort, shade provision emerges as one of the most effective welfare interventions available for beef cattle.

## 1. Introduction

Heat stress affects billions of domestic ruminants worldwide, including cattle, buffalo, sheep, and goats, with the greatest impacts occurring in tropical and subtropical regions where most of the world’s livestock are raised [1,2,3]. These animals face heat loads that regularly exceed their heat dissipation capacity for extended periods, triggering autonomic and behavioral responses that compromise welfare, health, and productivity. The impacts cascade through multiple systems: heat stress reduces feed intake, leads to digestive problems, causes immunosuppression and higher infectious disease risk, as well as higher mortality [4,5,6,7,8]. In tropical and subtropical regions, heat stress represents a chronic, pervasive challenge that animals must endure for much of their lives. Accordingly, production losses from heat stress in cattle were forecasted to amount to approximately $15 to 40 billion per year by the end of the century [1].

South America’s beef industry illustrates this challenge, with over 380 million beef cattle raised predominantly in pasture-based systems across Brazil, Argentina, Colombia, Paraguay, and Uruguay [9]. The region has become central to the global beef supply, accounting for nearly 30% of world production and over 40% of exports [9]. Production occurs across diverse climatic zones—from the Amazon margins to the Brazilian Cerrado and Colombia’s tropical lowlands—where cattle experience heat stress for extended periods annually. Although much of production is dominated by a predominance of *Bos indicus* (Nelore) cattle and their crosses—tropical-adapted breeds with efficient sweating mechanisms, lighter coat colors, and lower metabolic heat production [6,10,11,12]—these adaptations are often insufficient to prevent the many welfare challenges experienced under the extreme thermal conditions that characterize much of the region’s production areas, where radiant mean temperatures regularly exceed 40 °C and humidity compounds the thermal burden [13].

Thermal stress indices translate local weather variables into a scalar proxy for heat load. For example, the Temperature–Humidity Index (THI) combines air temperature and humidity into a single value, while more comprehensive indices such as the Comprehensive Climate Index (CCI) also incorporate wind speed and solar radiation [14]. Production studies quantify economic impacts through reduced feed intake, lower growth rates, decreased yield, and elevated mortality [15]. Biological indicators–cortisol concentrations, rectal temperatures, respiratory rates, heat shock proteins—track physiological responses as proxies for welfare states [16]. However, these approaches are limited for establishing the animal welfare burden of heat stress. First, they capture environmental conditions or acute responses at specific moments rather than cumulative impacts, missing how repeated or persistent thermal challenges accumulate into an overall welfare burden. Yet for welfare, duration matters: an animal experiencing moderate thermal discomfort for 5 h undergoes a different welfare burden than one experiencing the same intensity for 10 days. Second, they do not capture the impact as experienced by the animal, that is, the un-pleasantness or distress associated with heat exposure, which lies at the core of animal welfare. Moreover, none quantifies welfare impact in units that enable meaningful comparison with other metrics. Current indicators tell us when conditions are stressful or how animals respond physiologically, but provide no common currency for evaluating the magnitude of welfare impacts alongside that of economic costs and benefits. Without commensurable welfare metrics, it is impossible to assess trade-offs between welfare interventions and their implementation costs, or to compare the welfare impacts of different production decisions.

Addressing this quantification gap requires a standard means of characterizing exposure to heat stress, both acute and chronic, in a quantitative way, as well as a framework to translate thermal exposure into thermal discomfort actually experienced by animals, expressed in units that allow comparison with economic costs and other metrics.

The Welfare Footprint Framework (WFF) provides this final translation, expressing welfare impacts as cumulative time spent in affective states of varying intensities [17]. Rather than relying on proxy indicators of welfare or abstract scoring systems, the framework directly estimates the time animals spend in different affective states—providing a biologically meaningful, comparable and intuitive metric of welfare impact. The approach has been applied in diverse contexts [18,19,20,21,22] enabling, among other uses, the quantitative assessment of welfare trade-offs [19] and the cost-effectiveness of different practices and systems [18,20].

In this study we applied the WFF to quantify the welfare impact of thermal stress across a major South American beef production chain, and evaluated the cost-effectiveness of shade provision as a mitigation strategy, with economic returns estimated conservatively using finishing-phase performance da-ta. To this end, we characterize thermal conditions across 636 municipalities in Brazil, Argentina, Colombia, Paraguay, and Uruguay, drawing on five years of daily climate data (2019–2023) across tropical, subtropical and temperate zones. Both acute heat stress episodes and chronic exposure to thermal stress are considered. Chronic thermal stress is measured through a novel metric, the Annual Thermal Load (ATL), which integrates both the frequency and magnitude of daily excesses of a threshold of thermal comfort over time, capturing how thermal burden accumulates over time. We then applied the WFF to translate exposure to different thermal scenarios into time experienced in thermal discomfort of varying intensities. By quantifying the welfare impact of heat stress, this study identified critical regions where beef cattle face the most severe welfare burdens and evaluated the welfare gains achievable through mitigation, providing producers with evidence-based tools for improving animal welfare in a cost-effective manner, supply chain partners with priority areas for intervention, and policymakers with quantitative data to inform welfare standards.

## 2. Methods

This study quantified the welfare impact of heat stress across 636 municipalities where beef cattle farms in the Minerva Foods supply chain are located, distributed across major beef production areas in Brazil, Argentina, Colombia, Paraguay, and Uruguay. These locations encompass diverse climatic conditions from the Amazon margins to temperate grasslands.

South America’s beef cattle population exceeds 380 million head, with Brazil (234 million), Argentina, Colombia, Paraguay, and Uruguay representing approximately 25% of the global cattle inventory [9]. In contrast to North American and European systems, South American beef cattle are raised predominantly on pasture throughout their lives; even beef cattle that undergo feedlot finishing—approximately 17%—are confined only during the final 90–120 days before slaughter. Thus, the vast majority of animals’ cumulative lifetime thermal exposure occurs in extensive pasture systems. The analyses are representative of beef cattle production practices in the region, and are directed at animals that spend most of their time outdoors, exposed to climatic variations. We consider a baseline scenario where access to natural or artificial shade that is consistently effective for heat stress mitigation is absent. Although animals are raised with freedom of movement, and they will naturally seek shade, it is assumed that the availability, quality, and area of shade is insufficient before deliberate implementation of mitigation strategies. Additionally, the baseline scenario assumes the absence of widespread use of active cooling measures, such as forced ventilation systems common in confinement systems, or extensive use of water sprinklers in pastures. Finally, the study takes into account the intrinsic biological traits of the prevailing beef cattle breed, Nelore, known for its adaptability to tropical environments.

### 2.1. Climate Data and Heat Stress Assessment

Daily climate data spanning five years (2019–2023) were extracted from NASA’s Prediction of Worldwide Energy Resources (POWER) database at 0.5° × 0.5° spatial resolution. A five-year period (2019–2023) was analyzed to account for interannual climate variability and obtain representative thermal profiles for each location. For each of the 636 locations, geographic coordinates were determined using the centroid of the municipality where farms are located. Climate data were obtained at the municipality level for all farms, with the following meteorological variables extracted as daily averages: air temperature (°C), relative humidity (%), wind speed at 2 m height (m/s), and downward shortwave solar radiation flux (W/m^2^). Daily averages were used since hourly data were not available across all locations, and where available, the time series were often incomplete. This approach enabled comprehensive spatial coverage across all 636 municipalities. All data extraction was performed using Python (v 3.14, Python Software Foundation, Wilmington, DE, USA) scripts.

The Comprehensive Climate Index (CCI) [14] was selected to assess daily thermal stress intensity after careful evaluation of available indices. The Temperature–Humidity Index (THI), while widely used and simple to calculate, omits the effects of solar radiation and wind speed—factors that significantly modulate thermal load in open tropical environments. The Index of Thermal Stress for Cows (ITSC) [23], though biologically comprehensive, requires black globe temperature measurements, which are not directly available from the NASA POWER database, hence would require various modeling assumptions, introducing uncertainty. The CCI emerged as the optimal choice as it adjusts air temperature based on humidity, wind speed, and solar radiation effects, all variables available from NASA POWER. The CCI also applies to both hot and cold conditions, important here because the analysis spans tropical, subtropical and temperate (Argentina and Uruguay) climates. Indeed, the CCI has demonstrated superior predictive capacity for beef cattle welfare across diverse climatic conditions, as confirmed by recent validations in multiple geographic regions [24,25].

The CCI is calculated as CCI = T_a_ + FRH + FWS + FRAD, where T_a_ represents air temperature (°C) and F values adjustment factors. FRH adjusts for relative humidity effects, calculated using specific equations for humidity above and below 25%. FWS accounts for wind speed impacts on convective cooling, with different coefficients for temperatures above and below 27 °C. FRAD incorporates solar radiation load, with adjustments varying based on cloud cover and radiation intensity. All adjustment factors follow equations previously established and validated for beef cattle in extensive systems [14]. The CCI calculates “apparent temperature” in degrees Celsius, with six heat stress categories defined by specific thresholds [14]: (1) No stress (apparent temperature < 25 °C), representing the thermoneutral zone where energy expenditure for maintaining body temperature is minimal; (2) Mild stress (25–30 °C), associated with subtle physiological and behavioral adjustments including slight respiratory rate increases and greater shade-seeking behavior; (3) Moderate stress (30–35 °C), associated with significant physiological effort to maintain homeothermy, resulting in increased respiratory rate, pronounced peripheral vasodilation, active shade and water seeking, reduced feed intake, and decreased general activity affecting productive performance; (4) Strong stress (35–40 °C), expected to place thermoregulatory mechanisms under considerable pressure with pronounced behavioral alterations and significant production losses; (5) Extreme stress (40–45 °C), associated with dangerous body temperature increases with elevated risk of heat exhaustion, serious health problems, drastically reduced productivity, and potential mortality; and (6) Extreme danger (>45 °C), linked to high mortality risk even in healthy animals [26].

### 2.2. Chronic Heat Stress: Annual Thermal Load

The physiological impact of thermal stress on animals is determined not only by stress intensity in a single day, but by its duration and repetition over time. An isolated daily value does not indicate whether exposure to heat stress was a singular event or part of a sequence of thermally challenging days, nor does it capture history of the thermal stress the animal may have already accumulated. Although analyzing the frequency and duration of consecutive stressful day sequences is valuable for understanding critical event dynamics like heat waves, quantifying the total thermal load accumulated over an extended period can establish the global magnitude of thermal challenge imposed by the environment, reflecting the total physiological burden the animal must face to maintain homeostasis. It is typically the accumulation of wear, from total exposure to adverse conditions—whether a few days of extreme stress or many days of moderate stress—that can lead to chronic consequences in health, welfare, and performance.

In this context, the Annual Thermal Load (ATL) metric is defined as the sum of daily thermal stress excesses relative to a critical threshold, accumulated throughout a year:

$$\mathrm{Annual}\;\mathrm{Thermal}\;\mathrm{Load}=\overset{365}{\underset{d=1}{\sum }}max\;\left(0,\;CCI_{d}-{\mathrm{threshold}}_{d}\right)$$

where CCI_d_ represents the daily CCI value and a CCI = 30 °C marks the threshold for moderate stress onset (threshold *d*). This threshold corresponds to the boundary between ‘Mild’ and ‘Moderate’ stress categories in the CCI framework [14], representing the point at which significant thermoregulatory effort is required to maintain homeothermy. ATL captures the “excess thermal load” for each day. For instance, if CCI reaches 38 °C on a day, the excess thermal load for that day equals 8 °C in apparent temperature (38 °C–30 °C). Days with CCI below 30 °C contribute zero to the annual sum, as they fall within the thermoneutral zone. The 365 daily excess values are summed to obtain the annual thermal load for each location. The continuous nature of this metric preserves the magnitude of threshold excess, maintaining both the intensity and duration of thermal challenges experienced. This calculation was performed for each year from 2019 to 2023, then averaged arithmetically to derive the average ATL, accounting for interannual climate variability including extreme weather events. Locations were then classified into five categories of chronic thermal exposure risk based on their average ATL (Table 1), from Low to Extreme, representing levels of progressively compromised thermoregulatory capacity and physiological stress.

To characterize thermal exposure patterns within each ATL risk category, we analyzed how daily heat stress (measured by CCI) was distributed across all 636 locations. After calculating each location’s mean ATL and assigning it to one of five ATL risk categories, we counted how many days per year each location experienced at each CCI heat stress level (from “no stress” at CCI < 25 °C through “extreme danger” at CCI > 45 °C). These daily CCI stress counts were then averaged across all locations within the same ATL risk category. This produced representative profiles showing how daily heat stress patterns differed between ATL risk groups—for example, revealing whether locations with High ATL experience many days of moderate CCI or fewer days of severe CCI.

### 2.3. Heat Stress Scenarios

The welfare impact of heat stress depends not only on daily thermal intensity but critically on the chronic exposure context in which it occurs. A day where CCI is 35 °C represents different physiological and welfare challenges for beef cattle experiencing it as an isolated event compared to enduring it within months of accumulated thermal burden. While intuition might suggest animals chronically exposed to heat become “used” to it, evidence indicates that persistent exposure leads not to hedonic adaptation (where animals subjectively feel less discomfort over time) but to progressive depletion of compensatory capacity [27,28]. Behavioral responsiveness may diminish—reduced shade-seeking or postural adjustments—not from reduced perceived discomfort but from functional resignation or behavioral exhaustion. Conversely, animals from thermally neutral environments may display more pronounced acute responses to heat events, reflecting the contrast with their thermal baseline rather than greater subjective discomfort. Chronically exposed animals may thus experience equal or greater discomfort despite more muted responses.

To capture these context-dependent welfare impacts, we developed thirteen heat stress scenarios (Table 2) combining daily heat stress levels (CCI levels) with chronic exposure risk (ATL categories, Table 1). By modeling these scenarios separately, we aimed to capture how chronic exposure modulates acute stress responses, providing more accurate welfare quantification than approaches considering only daily maxima or annual averages.

### 2.4. Welfare Impact Quantification

The welfare impact of heat stress in each scenario from Table 2 was quantified using the Welfare Footprint Framework (WFF), a welfare assessment framework that expresses animal welfare impacts using a biologically meaningful and universal metric: cumulative time spent in affective states of varying intensities [17]. In this study, we focused on the direct thermal discomfort associated with heat stress—the negative affective state emerging from the inability to maintain thermal homeostasis. This assessment focuses specifically on the immediate subjective experience of being uncomfortably or dangerously hot, not on secondary consequences of heat stress such as the potential pain or discomfort from heat-induced diseases. The focus on direct thermal discomfort provides a conservative estimate of direct heat stress impacts, as the total welfare burden of heat stress encompasses these other secondary effects, extending beyond the immediate thermal experience.

For the quantification of welfare impacts using the WFF each daily episode of thermal discomfort was first divided into temporal segments based on factors likely to affect the perceived intensity of discomfort from heat stress over the day [18,21,22]. Segment boundaries were based on existing knowledge on biological processes (such as the shift from acute thermal challenge to metabolic exhaustion), behavioral transitions (cessation of active cooling attempts when exhaustion occurs), or external events (reduction in ambient temperature). For heat stress, the segmentation is critical because the perceived intensity of thermal discomfort is likely to change as animals progress through distinct physiological phases—from initial mobilization of compensatory mechanisms, through sustained thermoregulatory effort, to eventual recovery or exhaustion.

Next, for each temporal segment, two parameters were estimated: its duration (expressed as uncertainty ranges to account for both epistemic uncertainty and natural inter-individual variation) and the perceived intensity of the thermal discomfort during the segment. The WFF uses four intensity categories for negative states—Annoying, Hurtful, Disabling, and Excruciating—each operationally characterized by expected levels of functional disruption (Table S1) [17,21,22]. Existing evidence from multiple research areas was then reviewed, and compared with these operational criteria for each intensity, to inform the likelihood of thermal discomfort being more or less intense (i.e., the likelihood of each intensity category). Evidence typically includes (but is not limited to) behavioral patterns (e.g., posture changes, reduced activity, panting), neurophysiological measures (e.g., respiratory rate, core temperature), neurophysiological indicators, pharmacological responses, as well as evolutionary reasoning on the adaptive nature of heat stress responses. Unlike welfare assessment frameworks that force the choice of a single intensity category, the WFF assigns a probability for each intensity, reflecting both uncertainty and natural variation among individuals. Hypotheses about the intensity and duration of each temporal segment are then made explicit in a notation system, referred to as Pain-Track [21]. Finally, cumulative time in the negative state (i.e., thermal discomfort), also referred to as ‘Cumulative Pain’, is calculated by multiplying, for each segment, the probability assigned to each intensity by that segment’s duration, then summing across all intensities and segments (see [17,18,21]).

This framework was applied to estimate the cumulative time in thermal discomfort of each intensity per day under each of the thirteen scenarios in Table 2. To obtain annual impacts for each of the 636 locations, daily estimates were multiplied by the number of days per year that the location experienced each heat stress scenario.

### 2.5. Shade Mitigation Modeling

The primary benefit of shade is the drastic reduction in direct solar radiation incident on animals, one of the largest heat gain sources in hot, sunny environments. Additionally, shade promotes ground cooling beneath its projection, reducing heat radiated from the soil too. Evidence indicates that shade can reduce ground surface by 15 °C and radiant air temperature by up to 9 °C compared to direct sun exposure [29], depending on shade characteristics and local climate conditions. Accordingly, shaded cattle in their trial showed 5 °C lower body surface temperature, 10 breaths min^−1^ lower respiratory rate, and 3.4 L lower daily water intake compared to unshaded controls, alongside a 4.5% improvement in feed conversion [29].

The analysis assumes implementation of effective shade structures providing adequate coverage (3 m^2^ per animal) with materials that block solar radiation effectively. While various shade options exist (including lower-cost shade cloth), this analysis models properly designed structures that provide consistent, durable protection, as described previously [29]. The approach assumes that before implementation, animals have limited access to natural shade or that existing shade is insufficient in area, quality, or availability to effectively mitigate heat stress—a condition representative of most extensive beef production systems in South America where beef cattle are raised on open pastures.

The expected welfare improvements obtained through the provision of shade were calculated by re-categorizing heat stress days to lower CCI categories. Since CCI stress categories are defined by approximately 5 °C intervals, and evidence indicates a reduction in ground surface by 15 °C and radiant air temperature by up to 9 °C [21], we conservatively model shade provision as reducing thermal stress by one CCI category—an assumption that likely underestimates the actual benefits of effective shade structures. For example, if animals in a specific location experienced 100 days in the CCI category ‘Strong stress’ (35–40 °C), effective shade would result instead in 100 days of ‘Moderate stress’ (30–35 °C) during that period. This categorical reduction aligns with observed improvements in physiological indicators including reduced respiratory rates and core body temperatures under shaded conditions [29], as well as improvements in feed efficiency. The welfare impact of shade are quantified on an annual basis, reflecting cattle’s expo-sure whenever they are outdoors; economic valuation of these effects is addressed separately using finishing-phase data, as described next.

### 2.6. Economic Analysis

The economic impact of shade provision was calculated by comparing implementation costs against revenue gains from improved performance during the finishing phase, when performance data on shade effects in the region are available. Economic parameters were derived from Brazilian market conditions and the controlled trial data from Maia et al. [29], providing both welfare and economic outcomes for shade implementation in tropical *Bos indicus* production systems. The geographical overlap between the study location and a substantial fraction of our modeled locations enabled direct application of this empirically derived performance data.

The analysis was based on customized shade structures providing 3 m^2^ of shade per animal, following the design described by Maia et al. [29], with an initial investment ranging from US$75–93 per shaded spot. The proposed structure consisted of a steel roof coated with an aluminum–zinc–silicon alloy (55%, 43.5%, and 1.5%, respectively), while the anchoring system was constructed using steel rather than wood, as steel and wood have comparable costs in Brazil. The cost of raw materials used in the shade structure is variable, particularly for aluminum and steel, and may fluctuate by approximately 10–15% across the Brazilian territory and in comparison with neighboring countries, including Argentina, Paraguay, Uruguay, and Colombia [30]. A conservative 10-year lifespan was assumed for the economic calculation, though field evidence suggests these shade structures can last 15 years with minimal maintenance. The analysis assumed 3 finishing cycles per year (110–120 days each), standard for beef operations in the region. Per-animal shade cost was then obtained by dividing the investment by 30 cycles (10 years × 3 cycles/year).

Revenue gains were calculated using the 5 kg carcass weight differential reported by Maia et al. [29] and Edward-Callaway et al. [31] for *Bos indicus* × *Bos taurus* cattle (330 kg with shade vs. 325 kg without during finishing). For example, in the latter case, average daily gain (ADG), gain feed ratio (GF), and hot carcass weight (HCW) were significantly higher for shaded cattle: ADG (Shaded = 1.48 kg/animal/day; unshaded = 1.41 kg/animal/day; *p* < 0.001); GF (Shaded = 0.171; unshaded = 0.165; *p* < 0.01); HCW (Shaded = 354 kg; unshaded = 349 kg; *p* < 0.0001), with consistent results reported by Maia and colleagues [29]. This estimate was applied across all thermal load regions, though greater weight differentials might be expected in areas with more extreme heat stress, and in operations with less heat tolerant cattle phenotypes. The additional carcass weight was multiplied by average Brazilian beef prices (US$45–56 per arroba or US$3–4/kg, based on 2023–2024 market data) to determine gross revenue gains. Net economic outcomes were calculated by subtracting the amortized per-animal shade cost from the additional revenue. The cost-effectiveness of shade as a welfare intervention was then established by relating the economic investment to the reduction in hours of intense thermal discomfort achieved through shade provision. Revenue gains are highly conservative since they rely on improved performance during the finishing phase only.

## 3. Results

### 3.1. Heat Stress Exposure Patterns

**Figure 1:**
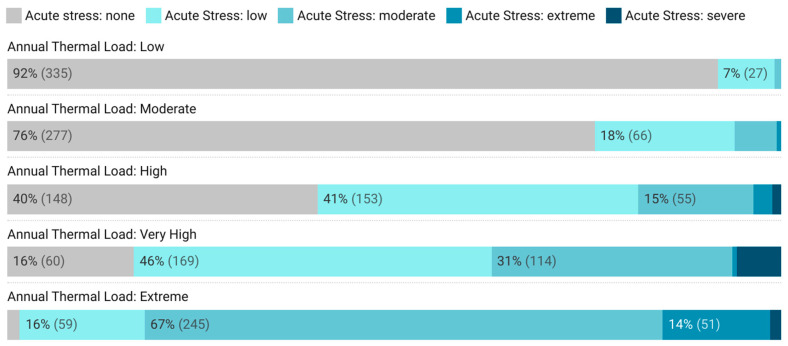
Days per year in different CCI categories classes (represented by colors, from gray to dark blue) for locations in different classes of chronic exposure to heat stress (Annual Thermal Load), from Low Chronic Thermal Load (upper bar) to Extreme Chronic Thermal Load (bottom bar).

The analysis of daily heat stress distribution across the 636 locations revealed distinct thermal profiles for each Annual Thermal Load (ATL) category (Figure 1). Locations in ‘Low Chronic risk’ ATL (top bar in Figure 1) averaged 92% of the year (335 days) without thermal stress, while ‘Moderate Chronic Risk’ locations showed more regular (approximately 24% of the year) days with thermal challenges. The ‘High Chronic Risk’ category, in turn, marked a critical threshold where beef cattle experienced some level of thermal stress for most (>60%) of the year. ‘Very High’ risk locations were associated with further thermal challenges, and most critically, locations of ‘Extreme’ risk had beef cattle experiencing moderate to intense stress most of the year. Accordingly, monthly temperature averages (Figure S1) showed that ‘Very High’ and ‘Extreme’ risk locations were associated with high heat load constantly throughout the year (27–29 °C monthly averages), with peaks between August and October. These locations, primarily in tropical zones of northern and central-western South America, showed minimal seasonal variation, exposing beef cattle to continuous heat load.

Critical to understanding welfare impacts, humidity patterns (Figure S2) revealed why tropical regions face extreme risk. Locations with ‘Very High’, ‘Extreme’ and ‘Severe’ thermal exposure had temperatures above 27 °C with relative humidity exceeding 75% during November through April. Evaporative cooling through sweating and panting depends on vapor pressure gradients between the animal and environment [32]. High relative humidity reduces this gradient, drastically decreasing cooling efficiency. Even heat-adapted breeds experience significant thermoregulatory challenges when high temperatures (>30 °C) combine with elevated humidity (>70%) [33]. Under these conditions, cattle are forced to rely on behavioral adaptations such as shade-seeking and reduced activity [34].

### 3.2. Geographic Distribution of Thermal Risk

**Figure 2:**
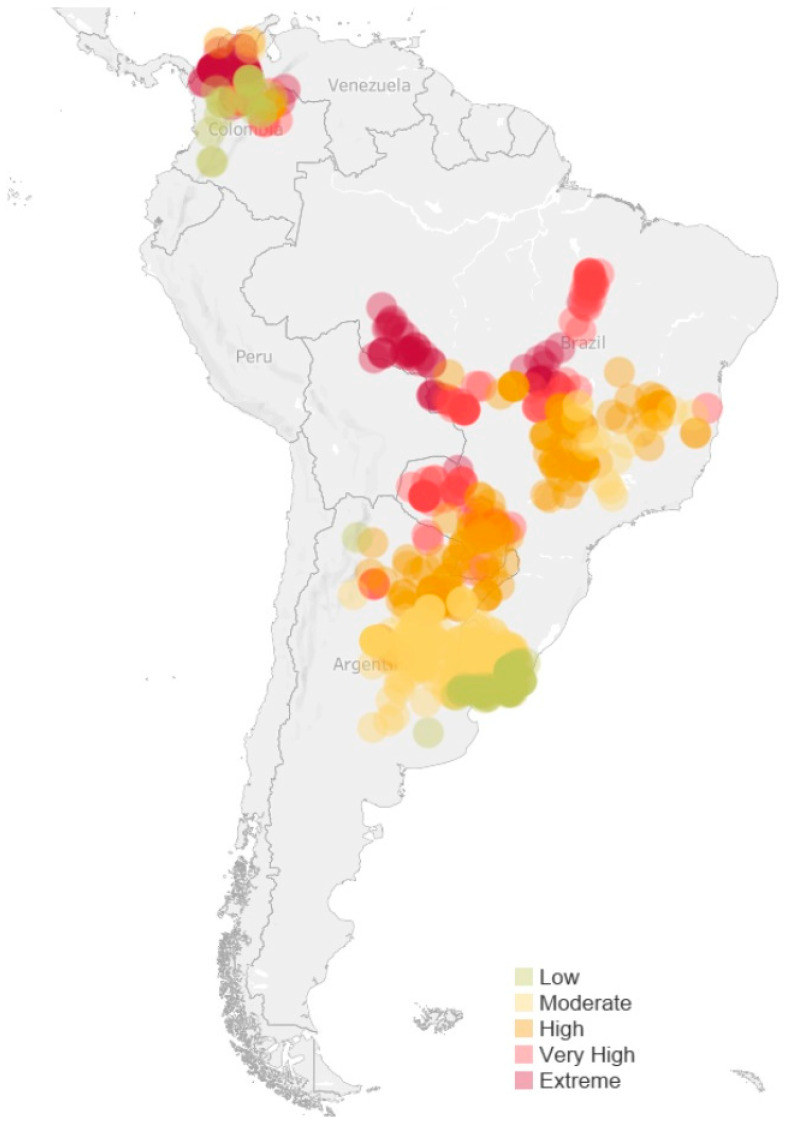
Distribution of locations in different classes of chronic annual thermal load (ATL). Colors: Low ATL = green; Moderate ATL = light yellow; High ATL = dark yellow; Very High ATL = orange; Extreme ATL = red.

Thermal risk patterns showed clear geographic clustering (Figure 2). Locations classified under ‘Extreme’ risk of chronic thermal stress (as measured by Annual Thermal Load) occurred in three main regions: northwest Colombia’s Caribbean lowlands, Brazil’s Rondônia state, and a continuous belt along the southern and southeastern Amazon margins encompassing northern Mato Grosso, eastern Acre, southern Pará, and northern Goiás. These regions share common features: year-round temperatures above 26 °C, high humidity (>70%), and minimal seasonal variation.

High and Very High thermal risk zones formed transitional areas around the regions of extreme risk. Moderate risk areas dominated central Brazil, including parts of the states of Minas Gerais, São Paulo, and Mato Grosso do Sul. Low risk locations are concentrated in Argentina’s Pampas, Uruguay, southern Paraguay, and Brazil’s southernmost states, where winters enable extended recovery periods.

When production volumes were incorporated (Figure S3), Rondônia emerged as a high priority region for intervention, combining extreme thermal risk and substantial production. Critically, 65% of beef cattle produced in this analysis experience ‘High risk’ of heat stress or above. Considering how representative these farms are of South American beef production, this indicates that heat stress is not only an occasional challenge but a chronic welfare burden for most beef cattle in the South American beef supply chain.

### 3.3. Quantification of Welfare Impacts

Heat stress patterns were translated into welfare impacts using the WFF, which quantifies welfare as cumulative time spent in affective states of varying intensity. Intensity categories are operationally defined by the degree of functional disruption they cause (Table S1). For each of the thirteen heat stress scenarios (Table 2), the framework involves (1) dividing each daily heat stress episode into temporal phases (here: initial stress, overload, recovery); (2) estimating the experienced intensity of thermal discomfort within each phase, based on existing evidence (e.g., behavioral, neurophysiological heat stress indicators); and (3) calculating cumulative time spent in thermal discomfort at each intensity, experienced daily and annually by beef cattle in regions with different chronic thermal risk profiles. The patterns emerging from implementing these steps are explored next.

#### 3.3.1. Duration of Thermal Discomfort from Daily Heat Stress Episodes

The total duration of each daily heat stress episode in each scenario was estimated (Table 3) by considering (1) how daily CCI values translate into hours per day spent above the thermal comfort threshold; (2) typical diurnal temperature variation, which vary from minimal (~3–6 °C) in tropical regions to substantial (~10–15 °C) in subtropical zones; and (3) whether nighttime conditions allow CCI to fall below the comfort threshold, enabling recovery. For instance, in tropical regions with extreme chronic thermal risk, beef cattle rarely experience relief from heat stress, often facing thermal discomfort continuously from early morning through late evening, as CCI rarely drops below the comfort threshold. In contrast, sub-tropical regions (predominantly low to moderate chronic thermal risk) concentrate heat exposure into shorter periods with longer nighttime relief.

Thermal discomfort from heat stress was then divided into three temporal segments: (I) initial stress, when thermal challenge begins and compensatory mechanisms are recruited; (II) overload, when sustained thermoregulatory effort reaches maximum capacity; and (III) exhaustion or recovery, when thermal load decreases and physiological responses begin returning toward baseline. This division aligns with the established two-stage model of stress response—acute and chronic phases—where the acute response involves rapid autonomic and voluntary activation lasting minutes to hours, while sustained exposure leads to exhaustion of compensatory reserves [35,36]. The initial mobilization phase is characterized by activation of peripheral thermoreceptors, triggering autonomic responses such as increased respiratory rate and peripheral vasodilation [35]. In Nelore, respiratory rate gradually increases from baseline (~25–35 breaths/min) as animals begin first-stage panting characterized by increased frequency but reduced tidal volume [37], with first-stage panting (40–80 breaths/min) transitioning to second-stage panting (>80 breaths/min) as heat load intensifies [38]. Core body temperature starts rising above the normal range (38–39.3 °C). *Bos indicus* cattle have a higher heat stress threshold than *Bos taurus* breeds, typically not showing marked responses until wet bulb temperatures exceed 30 °C [39]. As thermal exposure continues, animals may enter a state of greater thermoregulatory effort marked by metabolic shifts, reduced feed intake, and behavioral depression—indicators that compensatory mechanisms are becoming overwhelmed [40]. While Nelore cattle are more thermotolerant than temperate breeds, moderate chronic heat exposure reduces their compensatory capacity. Animals may transition to more pronounced panting, though the respiratory alkalosis seen in *Bos taurus* is typically less severe in *Bos indicus* [39]. Feed intake may also decline [40]. Water consumption increases, supporting evaporative cooling—a mechanism more efficient in Nelore due to their higher density of functional sweat glands [10]. The recovery phase involves gradual restoration of homeostatic balance as heat load decreases, with respiratory patterns typically returning to baseline before metabolic and endocrine function normalize [41]. In beef cattle, physiological indicators such as respiration rate and panting score serve as early warning signs of heat stress intensity.

Daily durations of thermal discomfort (Table 3) were then distributed across the three phases, for all heat stress scenarios (Table 4). The duration of each phase was estimated based on expected physiological effects in each case, such as how long compensatory mechanisms can be sustained before exhaustion, or how quickly recovery occurs given the degree of nighttime cooling.

In the Initial Stress phase (Phase I), scenarios with increasingly greater chronic heat exposure are assumed to progressively delay mobilization, reducing glucocorticoid receptor sensitivity and changing HPA axis responsiveness [42,43], hence delaying the onset of panting and sweating responses. Extreme acute temperatures may override these delays though, triggering immediate crisis responses.

Phase II (Overload) shows the greatest expansion—from 2–3 h to 6–7 h—partly due to longer environmental exposure but also because chronically stressed cattle may need to sustain compensatory efforts, such as panting, at reduced metabolic efficiency, requiring extended panting to achieve adequate cooling. Indeed, repeated heat exposure reduces feed intake and changes the energy metabolism, while sweating leads to electrolyte losses and reduced efficiency, forcing animals to maintain respiratory effort longer to achieve equivalent cooling. However, extreme chronic heat stress paired with extreme acute temperatures is expected to create a ceiling effect, with relatively shorter estimated Overload durations as evaporative cooling from the skin is unlikely to increase further. Additionally, second-stage panting increases alveolar ventilation, causing excessive CO_2_ expiration and respiratory alkalosis that may force earlier exhaustion [28].

Phase III (corresponding to ‘recovery’ in moderate scenarios but prolonged dysfunction in severe ones) is estimated to similarly extend with total duration. Moderate chronic scenarios are likely to allow recovery, while extreme chronic heat exposure is estimated to extend this period if repeated heat stress causes cellular and molecular responses including altered HSP70 expression that affects protein folding and cellular integrity [44,45]. In such cases, animals may not restore normal function even as temperatures decline, consistent with physiological exhaustion and documented mortality increases during heat waves [26].

Daily durations of thermal discomfort (Table 3) were then distributed across the three phases, for all heat stress scenarios (Table 4). The duration of each phase was estimated based on expected physiological dynamics under heat stress, such as how long compensatory mechanisms can be sustained before exhaustion, or how quickly recovery occurs given the degree of nighttime cooling [42].

In the Initial Stress phase (Phase I), scenarios with increasingly greater chronic heat exposure are assumed to progressively delay mobilization, reflecting a change in HPA axis responsiveness and lower glucocorticoid receptor sensitivity [43,44], hence delaying the onset of panting and sweating responses. Under extreme acute temperatures, however, these delays may be overriden, triggering immediate crisis responses.

Phase II (Overload) lasts longer in scenarios with higher chronic thermal risk—from 2–3 h to 6–7 h—partly due to longer periods of severe environmental heat exposure and slower dissipation of heat under sustained thermoregulatory effort. Indeed, repeated heat exposure reduces feed intake and changes the energy metabolism, while sweating leads to electrolyte losses and reduced efficiency, forcing animals to maintain respiratory effort longer to achieve equivalent cooling. However, extreme chronic heat stress paired with extreme acute temperatures is expected to create a ceiling effect, with relatively shorter estimated durations as evaporative cooling from the skin is unlikely to increase further. Additionally, second-stage panting increases alveolar ventilation, causing excessive CO_2_ expiration and respiratory alkalosis that may force earlier exhaustion [28].

Phase III (corresponding to ‘recovery’ in moderate scenarios but prolonged dysfunction in severe ones) is estimated to last longer as total daily heat stress duration in-creases. Moderate chronic scenarios are likely to allow recovery, while extreme chronic heat exposure is estimated to extend this period if repeated heat stress causes cellular and molecular disruption, including altered HSP70 expression affecting protein folding and cellular integrity [45,46]. In such cases, animals may not restore normal function even as temperatures decline, consistent with physiological exhaustion and document-ed mortality increases during heat waves [26].

#### 3.3.2. Intensity of Thermal Discomfort from Heat Stress Episodes

In the WFF, estimates on the perceived intensity of a negative experience (here the thermal discomfort in each heat stress phase) are informed by existing evidence, including indicators of behavioral disruption (e.g., suppressed voluntary activities, posture, specific behaviors), neurophysiological deviations from baseline (from mild autonomic activation to systemic dysfunction), pharmacological responses, and evolutionary reasoning on the adaptive value of aversive states of varying intensities. To illustrate the approach, consider the evidence reviewed for the M-M scenario [8,46,47,48,49,50,51,52], namely a day in moderate acute heat stress (CCI between 30–35 °C) in a region with moderate chronic heat exposure over the year, in Table 5. Evidence on the potential discomfort associated with the Initial phase of heat stress (Phase I) includes, for example, the frequent observation of the maintenance (although reduced) of grazing, intermittent shade-seeking, and respiratory rates rising to first-stage panting. During the ‘Overload Phase’, behavioral suppression typically becomes more pronounced, with cessation of voluntary activities, prolonged immobility, and second-stage panting, while cortisol elevation and core temperature will often rise, indicating more severe physiological disruption. Evidence in the ‘Recovery phase’ may include reduced, but still residual physiological changes, suggesting incomplete return to baseline. Each piece of evidence is rated for consistency with operational definitions of each intensity hypothesis in the WFF (Table S1). For example, the observation of reduced grazing is consistent with the operational criteria associated with the Annoying and Hurtful intensity categories, namely with a level of discomfort that disrupts but does not prevent essential functions. Similarly, the observation of respiratory rate increases to first-stage panting in Phase I indicates a physiological response consistent with the definitions of Annoying and Hurtful discomfort, although Disabling discomfort cannot be completely rejected.

Ratings in Table 5 are then used to estimate the probability of each category of thermal discomfort intensity (Pain-Track of Table 6A). This probabilistic approach explicitly acknowledges uncertainty in assessment and natural variation in how different individuals may perceive the discomfort. When evidence suggests equal consistency with multiple categories of thermal discomfort intensity, probabilities are divided equally between them rather than forcing an arbitrary choice of one intensity category. When evidence favors certain categories but cannot rule out others, higher probabilities are assigned to the better-supported intensity categories while maintaining non-zero probabilities for plausible alternatives. If an intensity category is rejected (R) by the available evidence for a given phase, it is assigned a probability of 0% for that phase; a single rejection is sufficient to exclude that intensity category. While the exact probability values are necessarily subjective, this probabilistic treatment of uncertainty is more rigorous than selecting a single intensity category, which effectively assumes 100% certainty for one category and 0% for all others.

The intensity patterns described for scenario M-M are expected to shift across the remaining twelve scenarios, with increasing chronic heat exposure associated with indicators that reflect more severe underlying states, hence a greater likelihood of thermal discomfort being perceived as more intense. Animals in high chronic exposure scenarios (VH, E) are expected to display more extreme behavioral and physiological responses to the same CCI temperatures, indicating greater physiological strain. Chronically heat-stressed animals are also more likely to show earlier behavioral collapse, more pronounced drooling, prolonged immobility, and failure to resume normal activities even during recovery. Even more severe indicators are expected under the extreme acute scenarios (E, ED), including respiratory rates, core temperatures, behavioral suppression with trembling or more exceptionally collapse, and absence of voluntary movement even when shade is available. The combination of extreme acute heat stress and extreme chronic exposure to heat (scenarios E-E, ED-E) is expected to yield the most severe observable indicators—animals with minimal responsive behavior to stimuli, critical physiological parameters despite attempted compensation, and signs of systemic failure such as muscle tremors and inability to stand. The descriptions of evidence underlying the estimates of the intensity of daily thermal stress discomfort corresponding to the additional heat stress scenarios are available in the Supplemental Material (Tables S2–S5).

#### 3.3.3. Cumulative Time in Thermal Discomfort

**Figure 3:**
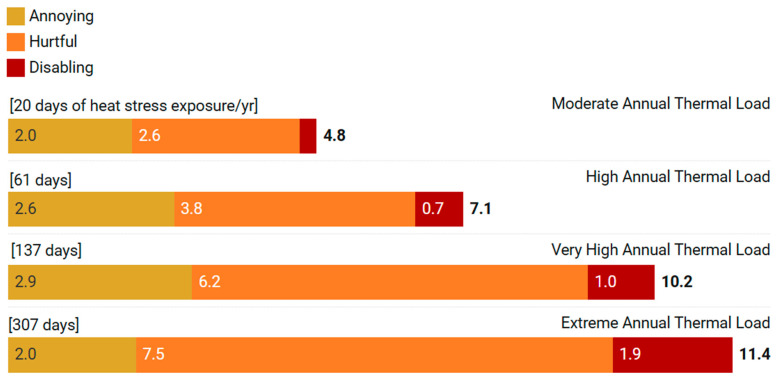
Daily thermal discomfort duration (hours per day) in beef cattle across regions with different annual thermal loads. Bars show average time spent in different discomfort intensities (Annoying, Hurtful, and Disabling) on heat stress days (CCI > 30 °C) for each thermal risk category (based on Annual Thermal Load; ATL). Numbers in brackets above the bars indicate the average number of heat stress days per year for animals located in the different ATL categories. Values represent mean daily discomfort when heat stress occurs, calculated from 636 locations across South America and thirteen heat stress scenarios modeled using the Welfare Footprint Framework.

Average daily time in each category of thermal discomfort are shown for locations in each category of chronic thermal risk in Figure 3. They were obtained by calculating how many days each location experienced each of the thermal stress scenarios annually, summing the corresponding daily time (hours) of thermal discomfort for the entire year, and dividing by the location’s annual heat stress days (CCI > 30 °C).

**Figure 4:**
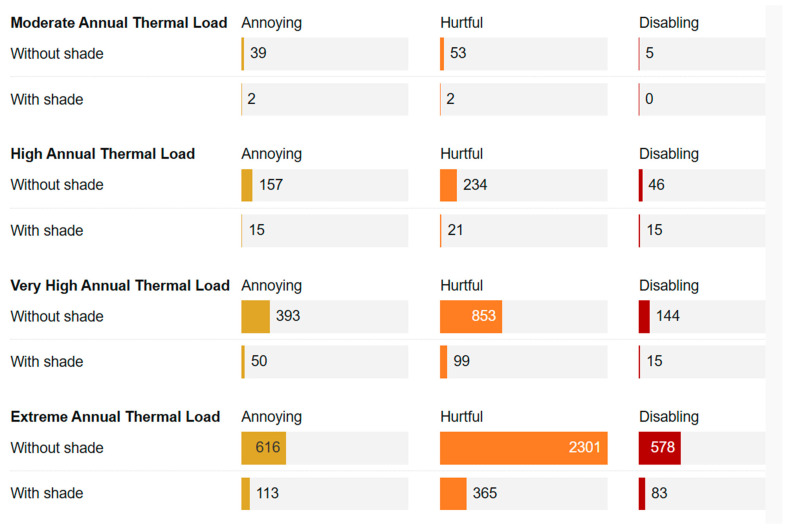
Hours per year, per animal, in thermal discomfort of different intensities in environments with and without effective shading in regions with different annual thermal loads.

The results reveal that beef cattle in moderate-risk regions (top bar in Figure 3) experienced discomfort of predominantly Annoying and Hurtful intensity during heat stress days (average 20 days per year). As chronic exposure increased, both the duration and intensity of thermal discomfort per daily heat stress episode increased, as shown by the progressively wider bars. Very high-risk areas (Very High ATL) reached 10.2 h of thermal discomfort daily, of which 1 h in Disabling intensity. Most critically, beef cattle in extreme-risk locations face heat stress over 300 days annually, experiencing on average 11.4 h of thermal discomfort per day, including nearly two hours in Disa-bling intensity, where thermoregulatory failure prevents normal behavior. These hours of Disabling thermal discomfort represent time during which the functional disruptions that define this intensity category are occurring, such as cessation of feeding and rumination, prolonged immobility, second-stage panting, along with other indicators listed in Table 5 and Tables S2–S5. Across regions with moderate, high, very high, and extreme annual thermal load, cattle without shade spent an average of 58, 280, 997, and 2879 h per year, respectively, in moderate to intense (Hurtful plus Disabling) thermal discomfort (Figure 4).

#### 3.3.4. Welfare Impact and Cost-Effectiveness of Shading

Figure 4 illustrates the welfare impact of shading for animals raised in locations with different thermal risk. Using the WFF, impact is represented as a reduction in the time spent in thermal discomfort per year, as achieved through effective shading. The welfare impact was most pronounced in extreme-risk regions, where shade reduced annual time spent in thermal discomfort of Disabling intensity from 578 to 83 h per animal (an 85% reduction in time spent in this most severe category of thermal discomfort). Similar patterns occurred for the other intensities of thermal discomfort, with combined reductions in time spent in thermal stress of Hurtful and Disabling intensity exceeding 3000 h annually. These estimates are very conservative, as they assume shade reduces thermal stress by only one CCI category.

The economic analysis focuses on the finishing phase (110–120 days) where weight gain is most pronounced and specific data on performance effects of shade are available. Economic parameters were derived from Maia et al. [29], who documented that *Bos indicus* cattle with access to customized shade structures (3 m^2^ per animal, US$80 initial cost, 15-year lifespan) gained 8 kg more hot carcass weight than unshaded controls during finishing in São Paulo state—a region classified as high thermal risk in our analysis. For conservative economic calculations, we assumed a 5 kg carcass weight differential applied across all thermal risk regions and local beef prices of US$46–56 per arroba. Since Maia’s benefits occurred in a high-risk region, performance improvements in very high and extreme risk areas—where beef cattle experience 3 to 5 more days in heat stress annually compared to high-risk regions—would likely exceed these estimates substantially.

The cost-effectiveness calculation for finishing operations reveals large returns. Assuming three finishing cycles annually, the per-animal shade cost over 10 years amounts to US$2.3 (30 cycles). The conservative 5 kg carcass weight gain generates US$15–19 in additional revenue per animal. After subtracting infrastructure costs, the net profit ranges from US$12–16 per animal, or US$12,000–15,700 per thousand animals finished, with a payback period of approximately four finishing cycles (16 months). These performance gains are assumed for the finishing phase only; lifetime shade pro-vision would likely yield substantially greater economic returns.

## 4. Discussion

This study presents the first quantitative assessment of welfare impacts from heat stress in beef cattle production. This study presents the first quantitative assessment of welfare impacts from heat stress in beef cattle production. By translating environmental conditions into cumulative hours of thermal discomfort of varying intensity we provide a biologically meaningful metric that captures both the duration and severity of heat stress under different environmental conditions and geographies. This approach enables a direct comparison of welfare impacts across regions, periods, and systems while enabling cost-effectiveness calculations for the implementation of different mitigation strategies, supplying producers with evidence-based tools for improving animal welfare. The magnitude of this burden, as estimated here, is substantial: cattle in high-risk regions experience approximately 280 h annually in moderate to intense thermal discom-fort, increasing to nearly 1000 h in very high-risk regions and exceeding 2800 h in extreme-risk regions. These figures position heat stress as likely one of the most significant welfare challenges faced by farmed animals globally, comparable in cumulative time to other major sources of pain and distress in intensive animal production [19,20].

The analysis of 636 locations across five countries revealed that heat stress is not an occasional challenge but the default condition for most South American beef cattle. This aligns with global assessments showing heat stress negatively affects billions of livestock worldwide [61], with particular severity in tropical regions [1]. With over 380 million beef cattle raised predominantly in extensive pasture systems across tropical and subtropical regions, the cumulative welfare burden is substantial. From a production perspective, prolonged heat exposure leads to reduced dry matter intake, decreased feed efficiency, and lower growth rates [53]. Reproductive performance also declines, as does immune function, increasing disease susceptibility [28]. In this analysis, the progression from manageable thermal challenge in moderate-risk regions to overwhelming physiological burden in extreme-risk areas illustrates how geographic location can drive these outcomes in tropical production.

The Annual Thermal Load metric introduced captures the accumulation of thermal burden. While a single day at 35 °C may seem manageable, experiencing such conditions 300 days annually depletes compensatory reserves and reduces resilience to additional stressors. Critically, the overlap between acute thermal intensity (high daily CCI) and chronic exposure (high annual thermal load) amplifies risk: animals already physiologically compromised cannot adequately mobilize compensatory mechanisms when under extreme heat episodes, increasing the probability of thermal exhaustion and, in severe cases, mortality. The geographic concentration of extreme risk in regions with high production volumes—Rondônia, southern Amazon margins, northwestern Colombia—make them priority targets for intervention.

One key intervention is the provision of shade [29,31,62,63,64], with meta-analytic evidence confirming that shaded cattle show improved weight gain, feed efficiency, and reduced respiratory rates [65]. The return from increased weight gain from properly designed shade structures exceeds implementation costs, effectively paying producers to improve welfare. The economic returns described compare favorably with other cost-benefit analyses, where it was estimated that heat stress costs the livestock industry billions annually, making mitigation investments economically justified [31,66,67].

While our economic analysis used Brazilian beef prices, producer prices vary across the region based on export market access, domestic consumption patterns, and regional supply factors. Uruguay and Paraguay typically have higher premiums due to quality-focused exports, while Colombia shows relatively lower prices. These variations affect absolute returns but not the fundamental cost-effectiveness of shade provision: even at the lower end of regional price ranges, the carcass weight differentials would generate returns exceeding implementation costs, while premium markets would yield proportionally higher returns. Similarly, material costs for shade structures may fluctuate by 10–15% across countries, but payback periods would remain under two years across plausible price scenarios.

The documented benefits of shade likely underestimate their impacts in extreme-risk regions where thermal stress is most severe. Additionally, our economic analysis focused only on the finishing stage; implementing shade throughout breeding and growing phases would likely multiply returns through improved conception rates, reduced calf mortality, improved growth, and reduced morbidity and mortality [31].

Successful implementation requires understanding shade as an essential infra-structure requiring proper engineering: structures must withstand tropical storms, pro-vide adequate height for convection, orient correctly for moving shadows, and last years with minimal maintenance [29]. Additionally, investigating breed-specific responses and integration with other cooling strategies could enhance effectiveness. For example, combining shade structures with forced ventilation (fans) has been shown to further improve weight gain and profitability in feedlot cattle [63]. Other practical interventions may also extend beyond shade provision to include improved access to water, management adjustments during peak thermal hours, incorporation of thermal criteria in spatial planning of grazing areas, and adoption of climate alerts for dynamic adjustments. For extensive pasture systems—where most South American beef cattle spend most of their lives—portable shade structures, strategic tree planting, or rotational grazing systems incorporating natural shade represent potential solutions, as recommended in heat stress management protocols.

Other mitigation strategies not addressed here include nutritional interventions such as micromineral supplementation. Although most research has focused on dairy cattle, recent evidence indicates that trace minerals including chromium, selenium, and zinc can enhance antioxidant defenses, reduce inflammatory responses, and improve thermoregulation under heat stress conditions [68]. These mechanisms are likely applicable to beef cattle and warrant investigation in extensive grazing systems.

Several limitations must be considered. Our analysis represents a sample of South American beef cattle production, and while comprehensive, it may not capture all regional variations. Additionally, knowledge remains limited regarding the precise temporal dynamics of thermal discomfort throughout the day, which led us to adopt conservative estimates of thermal discomfort duration and intensity. The duration of daily heat stress episodes was estimated based on typical diurnal temperature patterns, the dynamics of heat stress physiology, and climate characteristics of locations in different categories of chronic heat exposure. While this approach enabled assessment across hundreds of locations spanning diverse climatic zones, future applications for specific locations or farms could rely on hourly CCI measurements and direct behavioral and physiological observations, improving the precision of estimates. More critically, we did not quantify the mortality effects or secondary health consequences of heat stress, such as digestive disorders, immunosuppression, reproductive failures, all of which would likely substantially increase the welfare burden described. For example, chronic heat stress is a well-documented immunosuppressant in cattle [28]. Thermal stress activates the hypothalamic-pituitary-adrenal axis, resulting in elevated circulating cortico-steroids that inhibit inflammatory mediators and reduce immune function [28,43], in-creasing susceptibility to infectious diseases. Bovine respiratory disease (BRD), one of the most economically significant health problems in beef feedlots worldwide, including in South America, is explicitly recognized as a stress-related disease complex [69]. While our analysis focused on direct thermal discomfort, the immunosuppressive effects of heat stress likely compound the welfare burden through increased disease incidence and severity, further strengthening the economic and welfare case for heat stress mitigation.

## 5. Conclusions

The present analysis demonstrates that the cumulative welfare burden from heat stress, a challenge whose detrimental effects are well documented, can be quantified in units that enable direct cost-effectiveness assessment of interventions. Across 636 locations in five countries, this approach revealed that heat stress affects the majority of South American beef cattle for substantial portions of the year, providing quantitative evidence for the welfare and economic benefits of mitigation strategies, and the alignment of welfare improvements with profitability. For policymakers, this alignment provides quantitative grounding for the development of welfare standards informed by both welfare metrics and economic viability. This application of the Welfare Footprint Framework also provides a replicable framework for assessing the welfare burden of thermal challenges in other animal production systems. Where thermal challenges are present, shade provision should be understood not as an optional amenity but as a structural component of production in tropical and subtropical environments—essential infrastructure that simultaneously addresses animal welfare, productivity, and market access.

## Figures and Tables

**Table 1:** Classification of chronic thermal risk based on the average Annual Thermal Load index.

Risk Category	ATL (°C)	Description
Low	<100	Days exceeding threshold are rare or show minimal excess (e.g., ATL of 50 °C could result from 10 days at CCI 35 °C–5 °C excess daily or 50 days at 31 °C–1 °C excess daily). Animals experience minimal thermoregulatory challenges above stress thresholds.
Moderate	100–500	More frequent exposure to challenging thermal conditions. Animals regularly experience “moderate stress” days (30–35 °C) or fewer days at higher stress levels. Periods requiring active thermoregulation occur with some regularity.
High	500–1200	Considerable and frequent exposure to significant heat stress. Animals likely experience multiple days of “moderate to severe stress” (often >35 °C). For example, an ATL of 1000 °C could emerge from 100 days at CCI of 40 °C.
Very High	1200–2000	Substantial thermal load with frequent days of “severe stress” CCI level and some probable “extreme stress” days (>40 °C).
Extreme	>2000	Highest criticality level. Animals face prolonged “severe stress” periods and frequent “extreme stress” CCI or “extreme danger” CCI days, indicating chronic exposure to extremely adverse thermal conditions.

**Table 2:** Heat stress scenarios based on combinations of daily heat stress intensity (CCI categories) and chronic exposure context (Annual Thermal Load risk categories from Table 1). First letters = acute daily stress level from CCI (M = Moderate, S = Strong, E = Extreme, ED = Extreme Danger); second letters after hyphen = chronic risk level from ATL (M = Moderate, H = High, VH = Very High, E = Extreme). Each scenario represents the interaction between immediate thermal challenge and accumulated physiological burden.

CCI Stress Category	Thermal Risk Based on the Average Annual Thermal Load			
	Moderate Risk	High Risk	Very High Risk	Extreme Risk
Moderate (30–35 °C)	(1) M-M	(2) M-H	(3) M-VH	(4) M-E
Strong (35–40 °C)	(5) S-M	(6) S-H	(7) S-VH	(8) S-E
Extreme (40–45 °C)	—	(9) E-H	(10) E-VH	(11) E-E
Extreme Danger (>45 °C)	—	—	(12) ED-VH	(13) ED-E

**Table 3:** Estimated duration (hours) of daily thermal discomfort under the thirteen heat stress scenarios in Table 2, considering likely patterns of diurnal temperature variation, and regional climate where each scenario predominates. Ranges represent uncertainty and variability in expected duration. First letter: daily heat stress, CCI levels (M = Moderate, S = Strong, E = Extreme, ED = Extreme Danger); second letter: chronic risk, ATL categories (M = Moderate, H = High, VH = Very High, E = Extreme).

Scenario	Duration	Justification
(1) M-M	5–7	Locations predominantly in subtropical regions with moderate humidity and higher diurnal variation. CCI is expected to cross the 30 °C threshold from approximately 10–11 am to 4–5 pm when solar radiation peaks. Nights drop below threshold allowing recovery.
(2) M-H	7–9	Transitional tropical zones (Goiás, southern Cerrado) with increasing humidity. Higher humidity extends morning and evening discomfort periods. CCI expected to exceed 30 °C by ~9–10 am until 5–6 pm.
(3) M-VH	9–11	Northern Mato Grosso, Amazon edges. High humidity (>70%) means even 25–27 °C air temperature at 8 am produces CCI > 30 °C. Discomfort likely to persist until 7 pm despite moderate air temperatures.
(4) M-E	10–12	Scenario represents coolest days in extreme tropical zones. Even on moderate days, humidity > 75% and nighttime temperatures of about 24–25 °C mean CCI rarely drops below 30 °C. Nearly all daylight hours thermal stress.
(5) S-M	5–7	Regions with large diurnal variation allows brief but intense peaks. CCI reaches 35–40 °C for the afternoon period (12–5 pm) but substantial cooling at night.
(6) S-H	8–10	Humidity reduces nighttime cooling. CCI exceeds 35 °C from 10 am−6 pm with slower morning warming and evening cooling due to moisture.
(7) S-VH	10–12	High humidity throughout the day. Even at 8–9 am, temp of 28 °C + humidity + early sun produces CCI > 35 °C. Remains high past sunset.
(8) S-E	11–13	Common pattern in Rondônia. Minimal diurnal variation (nighttime CCI ~31–32 °C) means achieving a daily average of 35–40 °C requires nearly continuous elevation. Relief only in pre-dawn hours.
(9) E-H	7–9	Rare combination. When extreme days occur in high chronic areas, usually from dry heat waves allowing some nighttime recovery despite intense day stress.
(10) E-VH	9–11	Peak days in very hot regions. To average 40–45 °C requires sustained extreme conditions throughout daylight.
(11) E-E	10–12	Nighttime CCI remains >35 °C, daytime exceeds 45 °C. Daily averaging implies 10+ h in extreme range.
(12) ED-VH	8–10	Exceptional heat events. Despite catastrophic peaks, some diurnal variation still exists in VH regions, concentrating most severe stress in an 8 to 10 h window.
(13) ED-E	10–12	Extreme events in extreme regions. A daily average > 45 °C requires most of the day above this threshold. Nighttime may only drop slightly.

**Table 4:** Estimated duration (hours) of phases (I-III: Initial, Overload and Recovery, respectively) of daily heat stress episodes in the thirteen scenarios defined in Table 2. Ranges represent uncertainty and variability in average duration. For each scenario, the sum of durations across I–III corresponds to the total estimated duration of daily thermal discomfort re-ported in Table 3. First letter: daily heat stress, CCI levels (M= Moderate, S = Strong, E = Extreme, ED = Extreme Danger); second letter: chronic risk, ATL categories (M = Moderate, H = High, VH = Very High, E = Extreme).

Scenario	I	II	III	Justification
(1) M-M	1–2	2–3	1–2	Initial: Respiratory rate gradually increases from baseline to first-stage panting, core temperature rises. This mobilization was estimated at 1–2 h. Overload: sustained panting with increased water consumption and reduced feed intake before metabolic shifts occur. Recovery: as evening cooling begins, respiratory rate gradually decreases toward baseline
(2) M-H	1.5–2.5	3–4	2–2.5	Initial: Similar physiological progression but chronic exposure may blunt HPA response, delaying vasodilation, sweating. Overload: Reduced sweating may require maintaining panting longer to achieve similar cooling. Recovery: Higher cortisol and incomplete cooling extend recovery.
(3) M-VH	2–2.5	4–5	3–3.5	Initial: receptor downregulation expected to delay initial panting response and vasodilation. Overload: With sweat glands potentially lower efficiency, animals may need to sustain compensatory panting longer. Recovery: With nighttime CCI remaining high, only partial RR reduction is expected.
(4) M-E	2–3	5–6	3–3.5	Initial: Severe chronic exhaustion may maximally delay autonomic responses, longer to first-stage panting. Overload: Extreme depletion force prolonged low-efficiency compensation. Recovery: No return to baseline.
(5) S-M	1–1.5	2.5–3.5	1.5–2	Initial: Higher thermal gradient (CCI 35–40 °C) expected to trigger panting within 1–1.5 h. Overload: Second-stage panting with intact reserves. Recovery: Large evening temperature drop allows RR to normalize.
(6) S-H	1.5–2	4–5	2.5–3	Initial: Emergency panting response potentially delayed by chronic fatigue. Overload: Depleted reserves may require panting longer before exhaustion. Recovery: Smaller diurnal cooling prolongs high RR.
(7) S-VH	2–2.5	5–6	3–3.5	Initial: Despite strong stress, severe chronic fatigue may delay maximum panting. Overload: Near-maximal respiratory effort with compromised efficiency. Recovery: Minimal temperature relief means RR remains high.
(8) S-E	2.5–3	6–7	2.5–3	Initial: Extreme exhaustion may severely delay even emergency response. Overload: Prolonged struggle at minimal efficiency. Recovery: No respiratory normalization, only reduced panting
(9) E-H	0.5–1	3–4	3.5–4	Initial: Extreme heat may trigger crisis panting. Overload: Physiological ceiling reached more quickly but respiratory alkalosis may limit duration. Recovery: Cellular damage from extreme panting likely.
(10) E-VH	0.5–1	4–5	4.5–5	Initial: Immediate crisis response with maximum panting. Overload: ceiling-level panting despite alkalosis risk. Recovery: Severe physiological damage may prolong dysfunction.
(11) E-E	0.5–1	4.5–5.5	5–5.5	Initial: Despite exhaustion, life-threat likely triggers maximum panting quickly. Overload: Sustained at respiratory ceiling until exhaustion. Recovery: thermoregulatory failure maintains dysfunction.
(12) ED-VH	0.25–0.5	3–4	4.75–5.5	Initial: Extreme heat likely triggers immediate maximum response. Overload: Acute respiratory failure may limit active panting. Recovery: If survival occurs, critical dysfunction is expected
(13) ED-E	0.25–0.5	3.5–4.5	6.25–7	Initial: Immediate but potentially impaired crisis panting. Overload: Slightly prolonged by inability to mount full response. Recovery: Maximum thermoregulatory dysfunction.

**Table 5:** Summary of existing evidence [8,28,38,51,52,53,54,55,56,57,58,59,60] used to estimate the perceived intensity of the thermal discomfort experienced in each phase (I–III: Initial Stress, Overload and Recovery, respectively) of a daily episode of moderate heat stress in Nelore cattle (M-M scenario; Table 2), and ratings on consistency with the definitions of intensity (N: No discomfort, A: Annoying, H: Hurtful, D: Disabling, E: Excruciating; Table S1). Ratings are based on comparison between each piece of evidence and the criteria defined for each intensity category in Table S1. Ratings: evidence is ‘consistent’ (+), inconsistent (−) or ‘rejects’ (R) the intensity level, or consistency is unclear (?).

	Summary of Evidence	Intensity Hypothesis				
		N	A	H	D	E
I	Respiratory rate increases from baseline to first-stage panting	−	+	+	−	R
	Animals reduce but maintain grazing, seeking shade intermittently (CI)	−	+	+	R	R
	Core temperature rises above normal range (38.0–39.3 °C) (CI)	?	+	?	?	−
	Cortisol levels show initial elevation above baseline	−	+	+	?	?
	Animals maintain social interactions but reduce exploratory behavior (CI)	−	+	+	R	R
	From evolutionary perspective, moderate thermal challenge requires aversive signaling to motivate behavioral adjustments	−	+	+	+	−
II	Respiratory rate includes open-mouth panting	−	?	+	+	−
	Behavioral depression, cessation of voluntary activities. prolonged standing	R	−	+	+	−
	Core temperature rises even more above normal, approaching 41 °C	−	−	?	?	−
	Sustained cortisol elevation likely indicating severe physiological stress	−	+	+	?	−
	Drooling and signs of respiratory alkalosis from excessive panting	R	−	+	?	−
	Feed intake suppressed, water consumption typically increases	R	−	+	+	−
	Evolutionary perspective: prolonged, although moderate, thermal challenge requires aversive signaling	R	?	+	+	−
III	Respiratory rate gradually decreases but often still high (CI)	−	+	?	?	−
	Gradual resumption of grazing and social behaviors	−	+	?	R	R
	Core temperature slowly returns toward baseline	−	?	?	−	−
	Cortisol levels decline but may remain above baseline	?	+	?	?	−
	Residual metabolic disruption from lactate accumulation during overload (CI)	−	+	?	?	−

**Table 6:** (**A**) Pain-Track with hypotheses on how thermal discomfort intensity likely changes over time during a daily heat stress episode in Nelore cattle under the M-M scenario (moderate daily heat stress within moderate chronic thermal load). The vertical axis shows thermal discomfort intensities defined operationally by the Welfare Footprint Framework (Table S1). For each segment (I–III), estimated probability that beef cattle experience each intensity can be traced back to the estimates in Table 5. (**B**) Cumulative Pain table showing time in thermal discomfort at each intensity.

(A) Pain-Track		I. Initial Stress		II. Overload		III. Recovery	
Excruciating							
Disabling				10%			
Hurtful		30%		80%		10%	
Annoying		60%		10%		60%	
None		10%				30%	
Duration:		1–2 h		2–3 h		1–2 h	
**(B) Cumulative Pain**	**I. Initial Stress**		**II. Overload**		**III. Recovery**		**Cumulative Pain**
Excruciating							
Disabling			0.2–0.3 h				0.2–0.3 h
Hurtful	0.3–0.6 h		1.6–2.4 h		0.1–0.2 h		2–3.2 h
Annoying	0.6–1.2 h		0.2–0.3 h		0.6–1.2 h		1.4–2.7 h

## Data Availability

Climate data analyzed in this study are publicly available from NASA’s Prediction of Worldwide Energy Resources (POWER) database at https://power.larc.nasa.gov/ (accessed on 14 April 2025). The welfare impact results and thermal load calculations for all 636 locations are openly available at https://doi.org/10.17605/OSF.IO/SVFKQ. Location is anonymized to preserve the privacy of participating producers.

## Supplementary Materials

The following supporting information can be downloaded at: https://www.mdpi.com/article/10.3390/ani16020231/s1, Figure S1: Monthly average temperature by chronic thermal risk category; Figure S2: Monthly average relative humidity by chronic thermal risk category; Figure S3: Distribution of locations by thermal load with production volume; Table S1: Definition of negative affective experience intensities; Table S2: Summary of existing evidence used to inform the intensity of the thermal discomfort in beef cattle under moderate (30–35 °C) heat stress with increasing chronic annual thermal load risk; Table S3: Summary of existing evidence used to inform the intensity of the thermal discomfort in beef cattle under strong (35–40 °C) heat stress with increasing chronic annual thermal load; Table S4: Summary of existing evidence used to inform the intensity of the thermal discomfort in beef cattle under extreme (40–45 °C) heat stress with increasing chronic annual thermal load risk; Table S5: Summary of existing evidence used to inform the intensity of the thermal discomfort in beef cattle under extreme danger (>45 °C) heat stress with increasing chronic annual thermal load risk.
