# Supplementary material for: The Welfare Impact of Heat Stress in South American Beef Cattle and the Cost-Effectiveness of Shade Provision
Source: Animals (Basel). 2026 Jan 13;16(2):231. doi: 10.3390/ani16020231 (PMC12837348; doi:10.3390/ani16020231)
Supplement: Supplementary file 1 [file animals-16-00231-s001.zip › animals-4025843-supplementary.pdf]

Supplementary Material

Manuscript:

The Welfare Impact of Heat Stress in South American Beef Cattle and the Cost-Effectiveness of Shade Provision

Authors:

Cynthia Schuck-Paim, Wladimir J. Alonso, Anielly Freitas, Camila Pereira de Oliveira, Vinicius de Franca Carvalho Fonseca, Tâmara Duarte Borges

Table S1. Summary definition and expected observations in four categories of negative affective experience intensity (pain is used as a shorthand for any negative affective experience [1]).

Categories & Criteria	Annoying Pain	Hurtful Pain	Disabling Pain	Excruciating Pain
**Summary Definition**	Perceived only as a discomfort.	Pain intensity that clouds focus but allows basic tasks	Pain that takes priority over most bids for behavioural execution	Extreme level of pain felt unbearable even if very brief.
**Disruption of ability to conduct routine activities (1)**	No	To some degree	Substantial	Complete
**Performance of motivated & positive behaviours**	Not affected	Frequency and/or duration reduced	Not expected	Impossible
**Willingness to trade resources, work and safety (risk-taking) for pain relief**	Minimal	Moderate willingness to trade resources, risk-behaviour not affected	Willingness to trade important resources, work & safety to avoid pain	Complete focus on eliminating pain at all costs, including sacrifice of vital resources (eg life)
**Pain-specific manifestations (2)**	Not expected	Not expected, particularly in prey	Likely	Extreme
**Attention to pain: can pain sensation be ignored?**	Most of the time	For some time, depending on distraction	Very briefly, pain is continuous	No
**Attention to surroundings**	Not affected	Reduced to some degree.	Substantially reduced	Attention is exclusive to pain.
**Cognitively demanding tasks**	Not affected or mildly affected	Impaired	Substantially impaired	No cognitive task possible
**Departures of neurophysiological parameters from baseline**	Not expected	To some degree	Substantial	Substantial
**Are pain-relieving drugs effective?**	No effect expected.	Typical doses and drugs can eliminate the pain	Only higher doses or more powerful drugs	Powerful analgesics may only *reduce* pain.
**How long can pain be tolerated?**	Continuously	Continuously	Continuously, with major impairment of life quality	Can be willingly tolerated for only seconds to minutes

(1) Disruption of ability to conduct routine activities, such as eating, moving, foraging and exploring the environment; (2) e.g., vocalisations, shaking, muscle tension, specific motor patterns and facial expressions

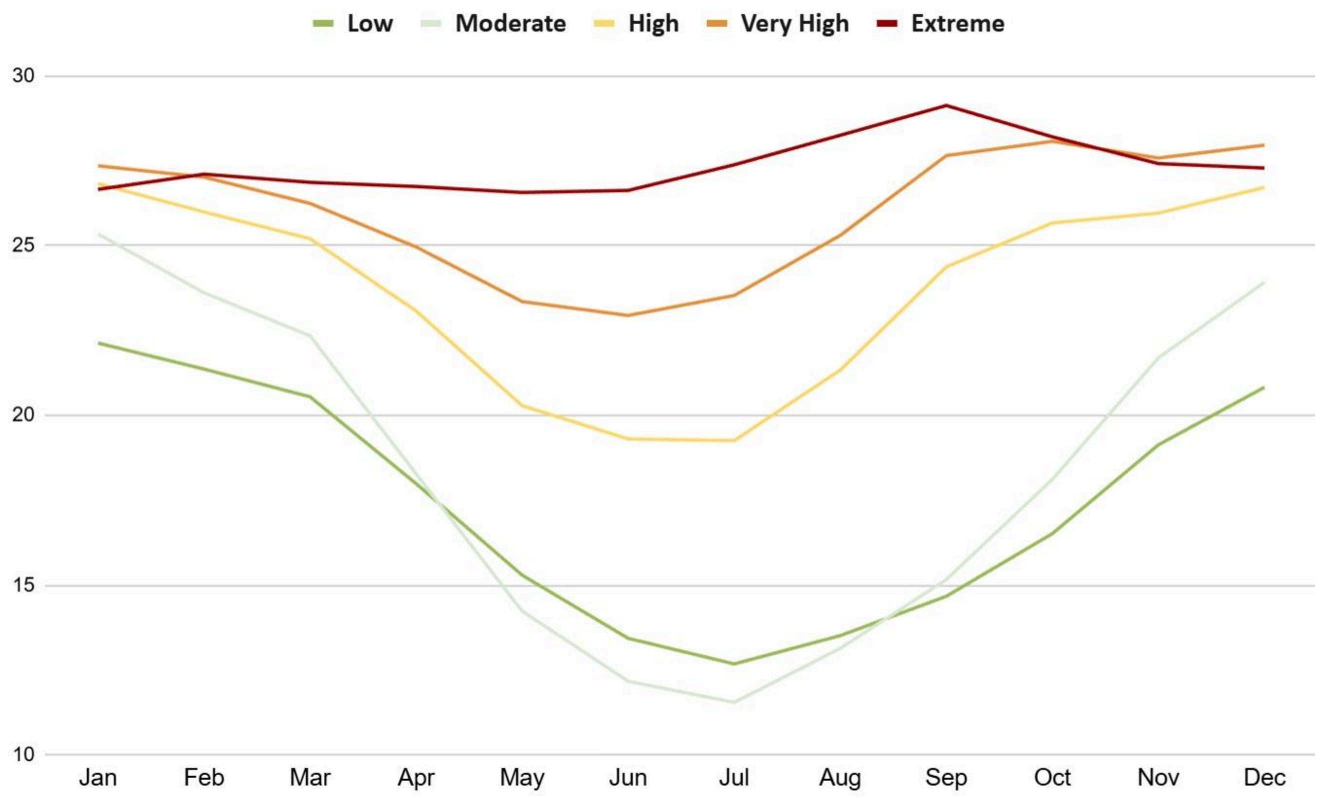

Figure S1. Monthly average temperature by chronic thermal risk category (Annual Thermal Load). Higher risk regions maintain high temperatures throughout the year, with lower seasonal variability.

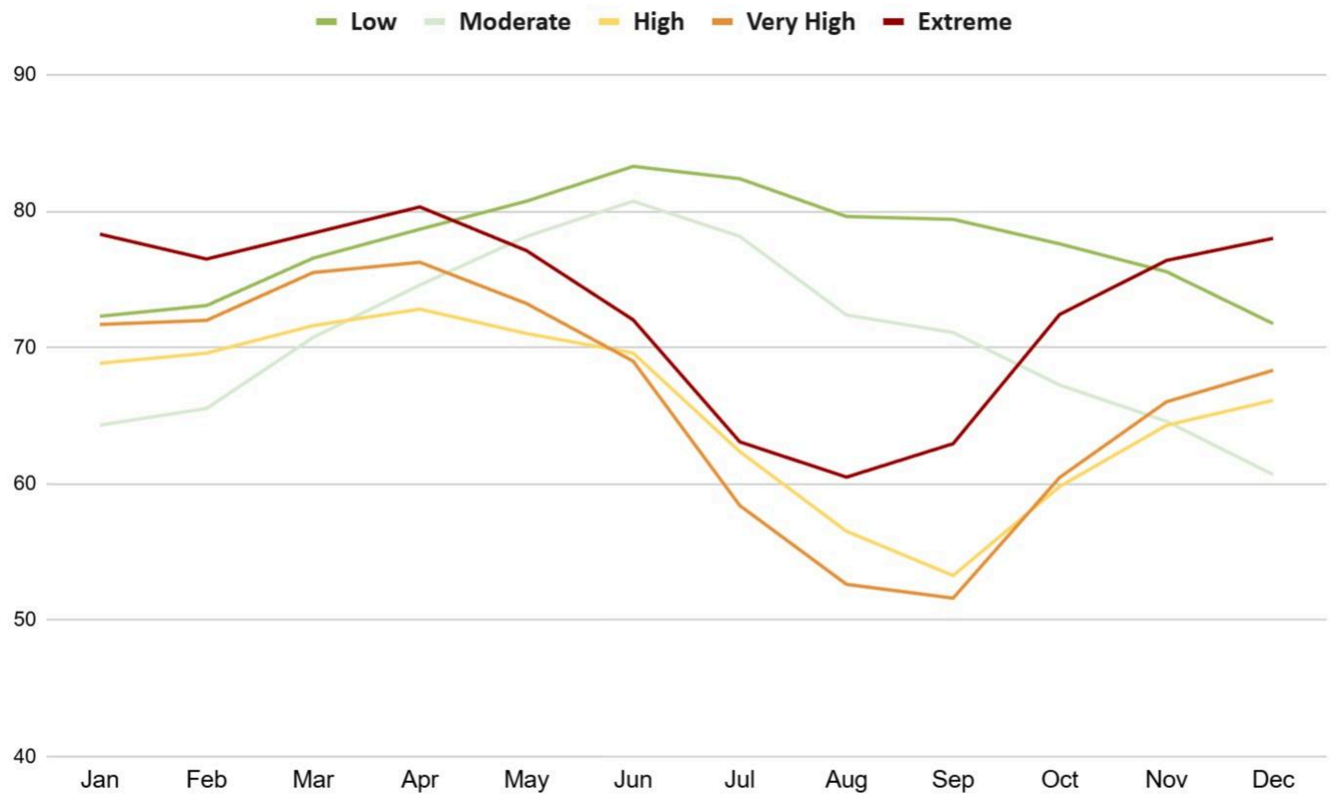

Figure S2. Monthly average relative humidity by chronic thermal risk category (Annual Thermal Load). Lower risk categories exhibit higher humidity in winter (June to August), typical in southern Brazil, Uruguay and Argentina.

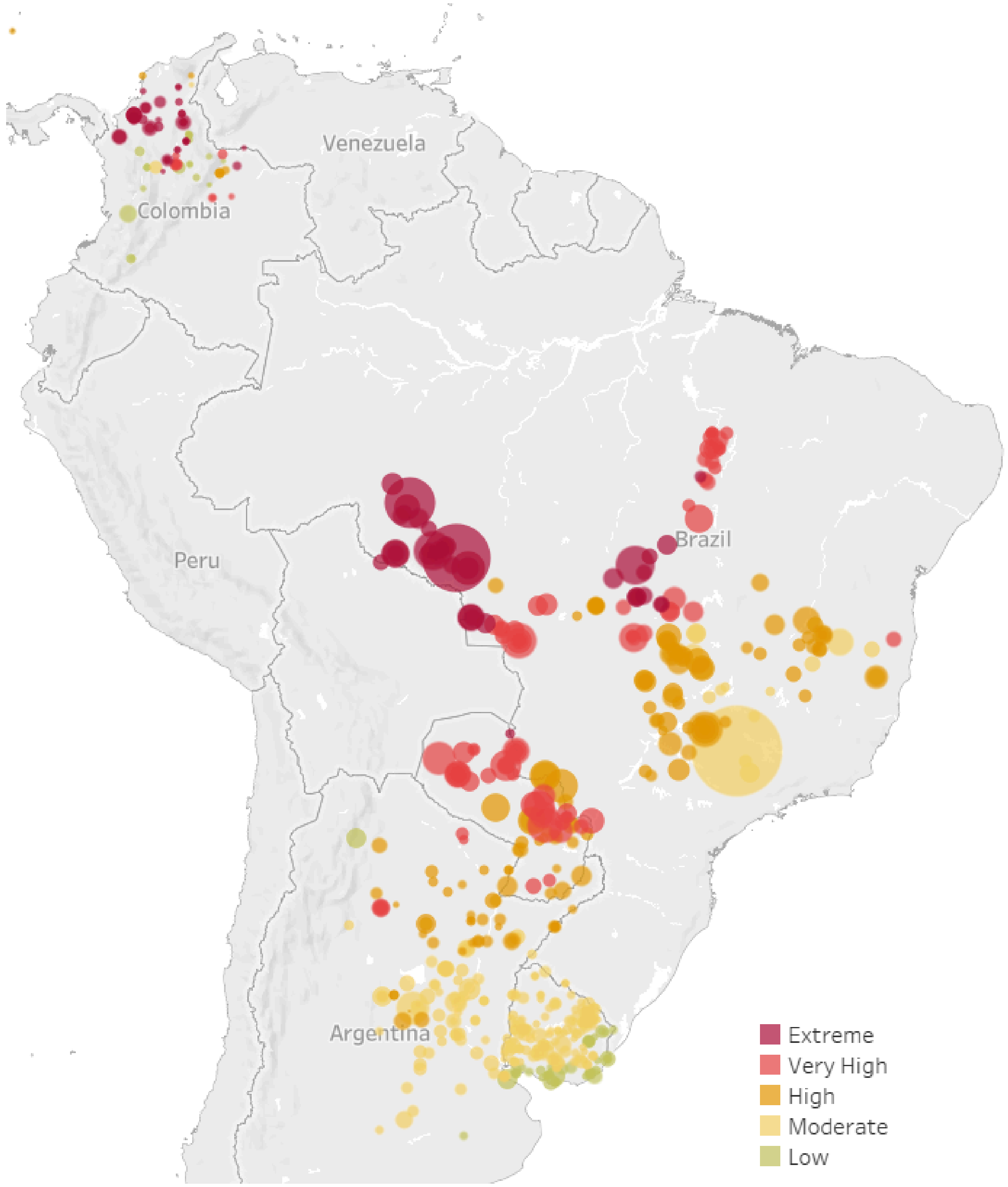

Figure S3. Distribution of locations in different classes of chronic annual thermal load, with marker size representing production volume.

Intensity of Thermal Discomfort from Daily Episode of Moderate Heat Stress (CCI:30-35°C) under different chronic annual thermal loads

Table S2. **Summary of existing evidence** [2–14] **used to inform the intensity of the thermal discomfort** in beef cattle under moderate (30-35°C) heat stress with increasing chronic annual thermal load risk, organized by phase of the daily heat stress episode: I (Initial Stress); II (Overload); III (Recovery).

	Marker	Moderate Heat Stress (CCI:30-35°C) in scenarios of different annual thermal load			
		Chronic risk: Moderate	Chronic risk: High	Chronic risk:Very High	Chronic risk:Extreme
I.	Resp. rate	Studies report gradual increases from baseline (around 25-35 breaths/min) to first-stage panting (40-60 breaths/min) under moderate heat exposure.	Here, responses may be blunted, potentially delaying the onset of panting	Receptor downregulation may further delay the initial panting response, consistent with differences in acute vs chronic heat stress.	Severe chronic exhaustion may lead to maximal delays in autonomic responses, resulting in longer times to reach first-stage panting.
	Grazing & Shade seeking	Animals may reduce but maintain grazing, with intermittent shade-seeking.	Reduced activity and initiation of feeding, potentially with more pronounced shade-seeking due to accumulated stress.	Animals may exhibit decreased exploratory behavior while continuing social interactions, with shade-seeking likely more frequent.	Grazing suppression may be more immediate, with shade-seeking behaviors potentially exhausted or less responsive.
	Core oC	Core temperature rises of 0.5-1°C above normal range (38.0-39.3°C).	Similar rises may occur, but with possible delays in thermoregulatory mechanisms like vasodilation.	Elevations may be sustained longer due to lower efficiency in cooling responses.	Extreme depletion may result in slower or less controlled temperature increases.
	Cortisol	Initial elevations above baseline have been reported, reflecting HPA activation.	Chronic exposure blunt HPA response, potentially causing lower/ delayed cortisol spikes.	Further downregulation may reduce cortisol response efficiency.	Severe exhaustion may minimize initial cortisol elevations.
	Social & Explorat. behavior	Animals may maintain social interactions but reduce exploratory behavior.	Social interactions may persist, but with greater reductions in exploration due to fatigue.	Decreased exploratory behavior may be more evident, while social interactions continue.	Exhaustion may limit both social and exploratory activities more substantially.
	Evolut.	Moderate thermal challenge may require aversive signaling to motivate behavioral adjustments.	Accumulated stress may alter signaling thresholds, potentially requiring stronger aversive cues.	Very high load may lead to habituation-like effects in signaling, though aversion persists.	Extreme load may overwhelm adaptive signaling, leading to resigned responses.

II.	Respiratory rate	Exceeds 80 breaths/min with open-mouth panting observed under sustained moderate stress.	Reduced sweat gland efficiency may require maintaining panting longer.	Animals may need to sustain compensatory panting longer due to lower efficiency.	Extreme depletion may force long low-efficiency compensation, possibly with irregular panting.
	Behavioral suppres.	Cessation of voluntary activities and prolonged standing.	Depleted reserves may extend periods of inactivity.	Near-maximal efforts may intensity suppression of voluntary activities.	Prolonged struggle at minimal efficiency may result in severe behav. depression.
	Core oC	Rises >1.5°C, approaching 41°C in moderate overload.	Incomplete cooling may sustain higher temperatures longer.	Minimal temperature relief may keep elevations persistent.	No effective cooling may lead to uncontrolled rises.
	Cortisol	Sustained elevations indicating physiological stress.	Higher cortisol may occur due to extended efforts.	Cortisol responses may be compromised but prolonged.	Depletion may limit sustained elevations, but stress remains.
	Drooling, resp. alkalosis	Signs from excessive panting, including drooling.	Alkalosis risk may increase with longer panting.	Compromised efficiency may heighten alkalosis.	Low-efficiency panting may exacerbate alkalosis and drooling.
	Feed & water intake,	Feed intake suppressed.	Suppression may be more prolonged due to fatigue.	Intake changes may be more severe with sustained efforts.	Extreme conditions lead to near-complete suppression.
	Evol	Prolonged moderate challenge may require aversive signaling.	Accumulated burden may intensify signaling for conservation.	High load may amplify aversion to motivate despite fatigue.	Overwhelmed systems may shift signaling toward survival resignation.
III.	RR	Gradually decreases but still high in some observations.	Smaller diurnal cooling may prolong high rates.	Only partial reduction expected with high nighttime CCI.	No respiratory normalization, only slight reductions.
	Grazing & social beh.	Gradual resumption as cooling begins.	Incomplete cooling delay resumption.	Minimal relief means limited resumption.	No return to baseline.
	Core oC	Slowly returns toward baseline in moderate recovery.	Higher residual heat may slow return.	Partial cooling may leave temperatures elevated.	No effective return, temperatures remain high.
	Cortisol	Decline but may remain above baseline.	Higher levels may persist longer.	Decline may be incomplete.	Minimal decline due to exhaustion.
	Metabolic disruption	Lactate accumulation from overload possible.	Extended recovery may increase disruption.	Severe damage may prolong metabolic issues.	Complete failure may lead to maximum disruption.

Translation of Evidence into Probabilities for Different Intensities

Pain-Track 2. Hypotheses on how thermal discomfort intensity changes during a daily heat stress episode in Nelore cattle in Scenario 2. The vertical axis shows thermal discomfort intensities defined operationally in Table S1. For each segment (I-III), estimated durations are justified in Table 3. Estimated probabilities can be traced back to Table S2.

	I. Initial Stress	II. Overload	III. Recovery	Cumulative Time in Thermal Discomfort
Excruciating				–
Disabling		20%		0.6-0.8
Hurtful	40%	70%	20%	3-4.3
Annoying	60%	10%	60%	2.1-3.4
None			20%	
	1.5 – 2.5 hrs	3 – 4 hrs	1.5 – 2.5 hrs	

Pain-Track 3. Hypotheses on how thermal discomfort intensity changes during a daily heat stress episode in Nelore cattle in Scenario 3. The vertical axis shows thermal discomfort intensities defined operationally in Table S1. For each segment (I-III), estimated durations are justified in Table 3. Estimated probabilities can be traced back to Table S2..

	I. Initial Stress	II. Overload	III. Recovery	Cumulative Time in Thermal Discomfort
Excruciating				–
Disabling		20%		0.8-1
Hurtful	50%	80%	40%	5.4-6.7
Annoying	50%		60%	2.8-3.4
None				
	2 – 2.5 hrs	4 – 5 hrs	3-3.5 hrs	

Pain-Track 4. Hypotheses on how thermal discomfort intensity changes during a daily heat stress episode in Nelore cattle in Scenario 4. The vertical axis shows thermal discomfort intensities defined operationally in Table S1. For each segment (I-III), estimated durations are justified in Tables 3 and 4. Estimated probabilities can be traced back to Table S2.

	I. Initial Stress	II. Overload	III. Recovery	Cumulative Time in Thermal Discomfort
Excruciating				–
Disabling		30%		1.5-1.8
Hurtful	60%	70%	60%	6.5-8.1
Annoying	40%		40%	2-2.6
None				
	2 – 3 hrs	5 – 6 hrs	3-3.5 hrs	

Intensity of Thermal Discomfort from Daily Episode of Strong Heat Stress (CCI:35-40°C) under different chronic annual thermal loads

Table S3. **Summary of existing evidence** [2–14] **used to inform the intensity of the thermal discomfort** in beef cattle under under strong (35-40°C) heat stress with increasing chronic annual thermal load, organized by phase of the daily heat stress episode: **I** (Initial Stress); **II** (Overload); **III** (Recovery).

	Marker	Strong daily heat stress episode (CCI:35-40°C) under different annual thermal load			
		Chronic risk: Moderate	Chronic risk: High	Chronic risk:Very High	Chronic risk:Extreme
I.	Resp. rate	Higher thermal gradient may trigger panting within shorter times, with increases to 60-80 breaths/min in strong heat.	Emergency panting response may be delayed by chronic fatigue, as in adaptive chronic exposures.	Despite strong stress, severe fatigue may delay maximum panting.	Extreme exhaustion may severely delay even emergency responses, resulting in longer times to effective panting.
	Grazing & Shade seeking	Rapid reduction in grazing, with active shade-seeking observed.	Fatigue may lead to more immediate cessation of activities.	Shade-seeking may be more urgent but less sustained.	Exhaustion may blunt behavioral responses.
	Core oC	Quicker rises of 1-1.5°C above baseline noted.	Delays in cooling may accelerate rises.	Lower efficiency may sustain faster elevations.	Severe delays may lead to uncontrolled increases.
	Cortisol	Rapid initial elevations due to stronger challenge.	Blunted HPA may reduce spikes.	Downregulation may further limit responses.	Exhaustion may minimize elevations.
	Social & Explorat. behavior	Quick reductions in exploration, social may persist briefly.	Fatigue may curtail social interactions sooner.	Severe load may suppress both more rapidly.	Extreme may limit all behaviors immediately.
	Evolut.	Strong challenge may elicit intense aversive signaling.	Chronic fatigue may modify signaling intensity.	High load may heighten aversion despite delays.	Extreme may overwhelm signaling mechanisms.
II.	Resp. rate	Second-stage panting with intact reserves observed.	Depleted reserves require panting longer before exhaustion.	Near-maximal effort with compromised efficiency.	Prolonged struggle at minimal efficiency.
	Behav. suppres.	More pronounced cessation,standing	Extended inactivity due to fatigue.	Suppression of activities more severe.	Severe depression from low efficiency.
	Core oC	Rises approaching 42°C.	Sustained higher due to incomplete cooling.	Persistent elevations with minimal relief.	Uncontrolled rises without cooling.
	Cortisol	Higher sustained elevations.	Prolonged due to extended efforts.	Compromised but extended.	Limited by depletion.

	Drooling, resp. alkalosis	Increased risk from intense panting.	Higher risk with longer durations.	Heightened symptoms from inefficiency.	Exacerbated by low efficiency.
	Feed intake	Marked suppression	More prolonged changes.	Severe alterations sustained.	Near-complete disruptions.
	Evol	Intense signaling to avert damage.	Amplified by accumulated burden.	Heightened aversion in high load.	Shift to survival modes in extreme.
III.	RR	Normalizes with large temp. drop.	Prolonged high rates from smaller cooling.	Remains high with minimal relief.	Only reduced panting, no normalization.
	Grazing & social beh	Quick resumption as cooling occurs.	Delayed by incomplete cooling.	Limited resumption.	Prevented by no baseline return.
	Core oC	Returns to baseline rapidly.	Slower return with residual heat.	Elevated due to partial cooling.	Remains high.
	Cortisol	Declines to baseline.	Persists higher.	Incomplete decline.	Minimal decline.
	Metabolic disruption	Minimal from brief overload.	Increased from extended phase.	Prolonged issues from damage.	Maximum from failure.

Translation of Evidence into Probabilities for Different Intensities

Pain-Track 5. Hypotheses on how thermal discomfort intensity changes during a daily heat stress episode in Nelore cattle in Scenario 5. The vertical axis shows thermal discomfort intensities defined operationally in Table S1. For each segment (I-III), estimated durations are justified in Table 3. Estimated probabilities can be traced back to Table S3.

	**I. Initial Stress**	**II. Overload**	**III. Recovery**	**Cumulative Time in Thermal Discomfort**
Excruciating				–
Disabling		25%		0.6-0.9
Hurtful	60%	75%	40%	3-4.3
Annoying	40%		40%	1-1.4
None			20%	0.3-0.4
	1-1.5 hrs	2.5 – 3.5 hrs	1.5 – 2 hrs	

Pain-Track 6. Hypotheses on how thermal discomfort intensity changes during a daily heat stress episode in Nelore cattle in Scenario 6. The vertical axis shows thermal discomfort intensities defined operationally in Table S1. For each segment (I-III), estimated durations are justified in Table 3. Estimated probabilities can be traced back to Table S3.

	I. Initial Stress	II. Overload	III. Recovery	Cumulative Time in Thermal Discomfort
Excruciating				–
Disabling		30%		1.2-1.5
Hurtful	80%	70%	60%	5.5-6.9
Annoying	20%		40%	1.3-1.6
None				
	1.5-2 hrs	4-5 hrs	2.5-3 hrs	

Pain-Track 7. Hypotheses on how thermal discomfort intensity changes during a daily heat stress episode in Nelore cattle in Scenario 7. The vertical axis shows thermal discomfort intensities defined operationally in Table S1. For each segment (I-III), estimated durations are justified in Table 3. Estimated probabilities can be traced back to Table S3.

	I. Initial Stress	II. Overload	III. Recovery	Cumulative Time in Thermal Discomfort
Excruciating				–
Disabling		40%		2-2.4
Hurtful	90%	60%	70%	6.9-8.3
Annoying	10%		30%	1.1-1.3
None				
	2 – 2.5 hrs	5-6 hrs	3-3.5 hrs	

Pain-Track 8. Hypotheses on how thermal discomfort intensity changes during a daily heat stress episode in Nelore cattle in Scenario 8. The vertical axis shows thermal discomfort intensities defined operationally in Table S1. For each segment (I-III), estimated durations are justified in Table 3. Estimated probabilities can be traced back to Table S3.

	I. Initial Stress	II. Overload	III. Recovery	Cumulative Time in Thermal Discomfort
Excruciating				–
Disabling	5%	45%		2.8-3.3
Hurtful	90%	55%	90%	7.8-9.3
Annoying	5%		10%	0.38-0.45
	2.5 – 3 hrs	6-7hrs	2.5-3 hrs	

Intensity of Thermal Discomfort from Daily Episode of Extreme Heat Stress (CCI:40-45°C) under different chronic annual thermal loads

Table S4. **Summary of existing evidence** [2–14] **used to inform the intensity of the thermal discomfort** in beef cattle under extreme (40-45°C) heat stress with increasing chronic annual thermal load risk, organized by phase of the daily heat stress episode: I (Initial Stress); II (Overload); III (Recovery).

	Marker	Extreme daily heat stress episode (CCI:40-45°C) under different annual thermal load		
		Chronic risk: High	Chronic Risk: Very High	Chronic Risk: Extreme
I.	Resp. rate	Extreme heat may trigger crisis panting quickly, reaching 100-120 breaths/min.	Immediate maximum panting despite risks, with potential for inefficiency in chronic contexts.	Despite exhaustion, life-threat may trigger maximum panting quickly.
	Grazing & Shade seeking	Immediate cessation, urgent shade-seeking.	More urgent but potentially impaired.	Blunted responses due to exhaustion.
	Core oC	Rapid rises >2°C reported.	Faster elevations with compromised cooling.	Uncontrolled due to severe delays.
	Cortisol	Sharp elevations from crisis response.	Limited by downregulation.	Minimized by exhaustion.
	Social & Explorat. behavior	Abrupt suppression of all activities.	More rapid curtailment.	Immediate limitation.
	Evolut.	Crisis signaling for immediate survival actions.	Amplified despite fatigue.	Overwhelmed in extreme load.
II.	Resp. rate	Physiological ceiling reached, >150 breaths/min possible.	Maintained at ceiling despite alkalosis risk.	Sustained until exhaustion at ceiling.
	Behav. suppres.	Complete cessation observed.	More severe suppression.	Prolonged struggle leading to depression.
	Core oC	Exceeds 42°C, risking exhaustion.	Persistent high with no relief.	Complete failure sustaining highs.
	Cortisol	Maximal elevations.	Compromised maximal.	Limited by depletion.
	Drooling, resp. alkalosis	High risk from extreme panting.	Heightened from sustained effort.	Exacerbated by inefficiency.
	Feed intake	Total suppression, extreme increase.	More severe disruptions.	Near-permanent in phase.

	Evol	Intense aversion to prevent fatal overheating.	Heightened in high load.	Survival resignation in extreme.
III.	RR	Still elevated from damage.	Remains high from severe damage.	Maintains high from failure.
	Grazing & social beh	Delayed by cellular damage.	Prolonged dysfunction limits.	Complete failure prevents.
	Core oC	Slow return with damage.	Elevated due to dysfunction.	No return.
	Cortisol	Persist above baseline.	Incomplete decline.	Minimal decline.
	Metabolic disruption	From extreme panting alkalosis.	Severe from prolonged.	Maximum from failure.

Translation of Evidence into Probabilities for Different Intensities

Pain-Track 9. Hypotheses on how thermal discomfort intensity changes during a daily heat stress episode in Nelore cattle in Scenario 9. The vertical axis shows thermal discomfort intensities defined operationally in Table S1. For each segment (I-III), estimated durations are justified in Table 3. Estimated probabilities can be traced back to Table S4

	I. Initial Stress	II. Overload	III. Recovery	Cumulative Time in Thermal Discomfort
Excruciating				–
Disabling	20%	35%		1.15-1.6
Hurtful	70%	65%	60%	4.4-5.7
Annoying	10%		40%	1.45-1.7
None				0
	0.5-1 hrs	3-4 hrs	3.5-4 hrs	

Pain-Track 10. Hypotheses on how thermal discomfort intensity changes during a daily heat stress episode in Nelore cattle in Scenario 10. The vertical axis shows thermal discomfort intensities defined operationally in Table S1. For each segment (I-III), estimated durations are justified in Table 3. Estimated probabilities can be traced back to Table S4

	I. Initial Stress	II. Overload	III. Recovery	Cumulative Time in Thermal Discomfort
Excruciating				–
Disabling	25%	40%	15%	2.4-3
Hurtful	75%	60%	75%	6.15-7.5
Annoying			10%	0.45-0.5
	0.5-1 hrs	4 – 5 hrs	4.5-5 hrs	

Pain-Track 11. Hypotheses on how thermal discomfort intensity changes during a daily heat stress episode in Nelore cattle in Scenario 11. The vertical axis shows thermal discomfort intensities defined operationally in Table S1. For each segment (I-III), estimated durations are justified in Table 3. Estimated probabilities can be traced back to Table S4

	**I. Initial Stress**	**II. Overload**	**III. No-recovery**	**Cumulative Time in Thermal Discomfort**
Excruciating				–
Disabling	30%	60%	30%	4.4-5.2
Hurtful	70%	40%	70%	5.7-6.8
Annoying				
	0.5-1 hrs	4.5-5.5 hrs	5-5.5 hrs	

Intensity of Thermal Discomfort from Daily Episode of Extreme Danger Heat Stress (CCI>45°C) under different chronic annual thermal loads

Table S5. **Summary of existing evidence** [2–14] **used to inform the intensity of the thermal discomfort** in beef cattle under extreme danger (>45°C) heat stress with increasing chronic annual thermal load risk, organized by phase of the daily heat stress episode: I (Initial Stress); II (Overload); III (Recovery).

	Marker	Extreme danger heat stress episode (CCI>45°C) under different thermal load	
		Chronic Risk: Very High	Chronic Risk: Extreme
I.	Respiratory rate	Catastrophic heat may trigger immediate maximum response.	Immediate but potentially impaired crisis panting.
	Grazing and shade-seeking	Instant cessation, desperate seeking.	Blunted due to maximal exhaustion.
	Core oC	Extremely rapid rises >3°C.	Uncontrolled from impaired responses.
	Cortisol levels	Immediate spikes if possible.	Minimized by severe exhaustion.
	Social, exploration behavior	Total immediate suppression.	No responses possible.
	Evol. perspective	Maximal crisis signaling for survival.	Overwhelmed signaling.
II.	Respiratory rate	Acute failure may limit active panting.	Slightly prolonged by inability to respond fully.
	Behavioral depression	Complete and potentially fatal.	Prolonged struggle to failure.
	Core temperature	Exceeds ~43°C, risking stroke.	Sustained fatal levels.
	Cortisol	Variable from failure.	Limited responses.
	Drooling, resp. alkalosis	Extreme risk from any panting.	Exacerbated by impairments.
	Feed intake	Absolute suppression.	Permanent disruptions.
	Evolutionary perspective	Aversion at lethal thresholds.	Resignation to failure.
III.	Respiratory rate	Critical dysfunction if survival.	Maximum dysfunction.
	Resumption of behaviors	Expected critical limitations.	No resumption.
	Core temperature	No quick return.	Remains dysfunctional.
	Cortisol levels	Persist irregularly.	No decline.
	Metabolic disruption	From acute failure.	Maximum from complete failure.

Translation of Evidence into Probabilities for Different Intensities

Pain-Track 12. Hypotheses on how thermal discomfort intensity changes during a daily heat stress episode in Nelore cattle in Scenario 12. The vertical axis shows thermal discomfort intensities defined operationally in Table S1. For each segment (I-III), estimated durations are justified in Table 3. Estimated probabilities can be traced back to Table S5.

	I. Initial Stress	II. Overload	III. No-recovery	Cumulative Time in Thermal Discomfort
Excruciating				–
Disabling	40%	60%	30%	3.3-4.2
Hurtful	60%	40%	60%	4.2-5.2
Annoying			10%	0.5-0.6
	0.25-0.5 hrs	3-4 hrs	4.75-5.5 hrs	

Pain-Track 13. Hypotheses on how thermal discomfort intensity changes during a daily heat stress episode in Nelore cattle in Scenario 13. The vertical axis shows thermal discomfort intensities defined operationally in Table S1. For each segment (I-III), estimated durations are justified in Table 3. Estimated probabilities can be traced back to Table S5

	I. Initial Stress	II. Overload	III. No-recovery	Cumulative Time in Thermal Discomfort
Excruciating				–
Disabling	50%	70%	40%	5.1-6.2
Hurtful	50%	30%	60%	4.9-5.8
Annoying				
	0.25-0.5 hrs	3.5-4.5 hrs	6.25-7 hrs	

References

1. Alonso WJ, Schuck-Paim C. Welfare Footprint Framework - Methodological Foundations and Quantitative Assessment Guidelines. Center for Welfare Metrics. 2025;Sao Paulo. doi:10.17605/OSF.IO/94BXS.

2. Gaughan, J.B.; Mader, T.L. Body temperature and respiratory dynamics in un-shaded beef cattle. *Int. J. Biometeorol.* **2014**, *58*, 1443–1450.

3. Idris, M.; Sullivan, M.; Gaughan, J.B.; Phillips, C.J.C. Behavioural responses of beef cattle to hot conditions. *Animals* **2024**, *14*, 2444.

4. Rashamol, V.P.; Sejian, V.; Bagath, M.; Krishnan, G.; Archana, P.R.; Bhatta, R. Physiological adaptability of livestock to heat stress: An updated review. *J. Anim. Behav. Biometeorol.* **2018**, *6*, 62–71.

5. Eckhardt, R.; Arablouei, R.; McCosker, K.; Bishop-Hurley, G.; Bagnall, N.; Hayes, B.; et al. Insights into thermal stress effects on performance and behavior of grazing cattle via multimodal sensor monitoring. *Sci. Rep.* **2025**, *15*, 27941.

6. Eckhardt, E.P.; Kim, J. Prolonged heat stress impact on molecular responses of skeletal muscle and growth performance in finishing beef steers. *J. Anim. Sci.* **2024**, *102*, 223–224.

7. Dos Santos, M.M.; Souza-Junior, J.B.F.; Dantas, M.R.T.; de Macedo Costa, L.L. An updated review on cattle thermoregulation: Physiological responses, biophysical mechanisms, and heat stress alleviation pathways. *Environ. Sci. Pollut. Res.* **2021**, *28*, 30471–30485.

8. Idris, M.; Uddin, J.; Sullivan, M.; McNeill, D.M.; Phillips, C.J.C. Non-invasive physiological indicators of heat stress in cattle. *Animals* **2021**, *11*, 71.

9. Shephard, R.W.; Maloney, S.K. A review of thermal stress in cattle. *Aust. Vet. J.* **2023**, *101*, 417–429.

10. Oke, O.E.; Eletu, T.A.; Akosile, O.A.; Fasasi, L.O.; Adeniji, O.E.; Ojedokun, M.Z.; et al. Behavioural adaptations of livestock to environmental stressors: Implications for welfare and productivity. *J. Appl. Anim. Res.* **2025**, *53*, 2583108.

11. Izquierdo, V.S.; da Silva Menezes, B.; Lopes, M.G.; Malaguez, E.G.; Lopes, F.; Pereira, F.M.; et al. Rumen-protected methionine modulates body temperature and reduces the incidence of heat stress during the hottest hours of the day in grazing heat-stressed *Bos indicus* beef cows. *Anim. Sci. J.* **2024**, *95*, e13980.

12. Beatty, D.T.; Barnes, A.; Taylor, E.; Pethick, D.; McCarthy, M.; Maloney, S.K. Physiological responses of *Bos taurus* and *Bos indicus* cattle to prolonged, continuous heat and humidity. *J. Anim. Sci.* **2006**, *84*, 972–985.

13. Mishra, S.R. Behavioural, physiological, neuro-endocrine and molecular responses of cattle against heat stress: An updated review. *Trop. Anim. Health Prod.* **2021**, *53*, 400.

14. da Silva, W.C.; da Silva, J.A.R.; Martorano, L.G.; da Silva, É.B.R.; de Carvalho, K.C.; Sousa, C.E.L.; et al. Thermal comfort of Nelore cattle (*Bos indicus*) managed in silvopastoral and traditional systems in a humid tropical environment in the Eastern Amazon, Brazil. *Vet. Sci.* **2024**, *11*, 236.
